# Macrophage migration inhibitory factor is critical for dengue NS1-induced endothelial glycocalyx degradation and hyperpermeability

**DOI:** 10.1371/journal.ppat.1007033

**Published:** 2018-04-27

**Authors:** Hong-Ru Chen, Chiao-Hsuan Chao, Ching-Chuan Liu, Tzong-Shiann Ho, Huey-Pin Tsai, Guey-Chuen Perng, Yee-Shin Lin, Jen-Ren Wang, Trai-Ming Yeh

**Affiliations:** 1 The Institute of Basic Medical Sciences, College of Medicine, National Cheng Kung University, Tainan City, Taiwan; 2 Department of Pediatrics, College of Medicine, National Cheng Kung University, Tainan City, Taiwan; 3 Department of Pathology, College of Medicine, National Cheng Kung University, Tainan City, Taiwan; 4 Department of Microbiology and Immunology, College of Medicine, National Cheng Kung University, Tainan City, Taiwan; 5 Department of Medical Laboratory Science and Biotechnology, College of Medicine, National Cheng Kung University, Tainan City, Taiwan; Icahn School of Medicine at Mount Sinai, UNITED STATES

## Abstract

Vascular leakage is one of the salient characteristics of severe dengue. Nonstructural protein 1 (NS1) of dengue virus (DENV) can stimulate endothelial cells to secrete endothelial hyperpermeability factor, macrophage migration inhibitory factor (MIF), and the glycocalyx degradation factor heparanase 1 (HPA-1). However, it is unclear whether MIF is directly involved in NS1-induced glycocalyx degradation. In this study, we observed that among NS1, MIF and glycocalyx degradation-related molecules, the HPA-1, metalloproteinase 9 (MMP-9) and syndecan 1 (CD138) serum levels were all increased in dengue patients, and only NS1 and MIF showed a positive correlation with the CD138 level in severe patients. To further characterize and clarify the relationship between MIF and CD138, we used recombinant NS1 to stimulate human cells *in vitro* and challenge mice *in vivo*. Our tabulated results suggested that NS1 stimulation could induce human endothelial cells to secrete HPA-1 and immune cells to secrete MMP-9, resulting in endothelial glycocalyx degradation and hyperpermeability. Moreover, HPA-1, MMP-9, and CD138 secretion after NS1 stimulation was blocked by MIF inhibitors or antibodies both *in vitro* and in mice. Taken together, these results suggest that MIF directly engages in dengue NS1-induced glycocalyx degradation and that targeting MIF may represent a possible therapeutic approach for preventing dengue-induced vascular leakage.

## Author summary

DENV NS1 induces endothelial glycocalyx degradation and hyperpermeability via HPA-1 and MMP-9 activation in an MIF-dependent manner.

## Introduction

Dengue virus (DENV) is a flavivirus that infects approximately 390 million people and causes 500,000 infections requiring hospitalization every year, with an associated mortality rate of 2.5% [1]. DENV infection usually causes a flu-like illness, known as dengue fever (DF), which is associated with high-grade fever and joint pain. Most dengue patients recover without hospitalization, but in some cases, patients develop potentially deadly complications called dengue hemorrhagic fever or dengue shock syndrome (DHF/DSS). According to the latest guidelines from the World Health Organization (WHO), dengue severity can be classified into dengue with or without warning signs and severe dengue. One of the main characteristics of DHF/DSS or severe dengue is plasma leakage. The increase in vascular permeability is the primary cause of plasma leakage, which finally causes hypotension and circulatory collapse. Because the mechanism underlying vascular hyperpermeability during DENV infection is not yet fully understood, and no specific approved treatments are available; only supporting treatments, such as fluid therapy, are available.

An increase in endothelial permeability is frequently associated with the degradation of the endothelial glycocalyx [2, 3]. Under normal physiological conditions, the glycocalyx acts as a barrier that controls numerous physiological processes, especially preventing the adhesion of leucocytes and platelets to the vessel walls [4, 5]. Degradation of the endothelial glycocalyx correlates to several vascular pathologies, including sepsis [6, 7]. Shedding of the endothelial glycocalyx is related to the activation of a heparan sulfate-specific heparanase, HPA-1 [5, 8]. Activated HPA-1 enhances shedding of the transmembrane heparan sulfate proteoglycan syndecan-1 (CD138) and elevates the level of CD138 in the bloodstream [7, 9, 10]. In addition, matrix metalloproteinases (MMPs) are capable of digesting many types of extracellular matrix, including the endothelial glycocalyx [11, 12]. Glycocalyx degradation is strongly associated with severe plasma leakage in dengue patients [13, 14]. However, the mechanisms causing glycocalyx degradation during DENV infection are not fully understood.

Recently, DENV nonstructural protein 1 (NS1) was found to play an important role in the pathogenesis of DENV-induced vascular leakage [15–17]. In addition, NS1 can induce the expression and activation of HPA-1, leading to endothelial glycocalyx degradation and hyperpermeability [18]. In our previous study, we found that DENV NS1 can increase vascular permeability through macrophage migration inhibitory factor (MIF)-induced autophagy [19]. MIF is a chemokine-like inflammatory cytokine that binds to cell surface receptors (CD74 and/or CXCR2/4/7) and activates downstream signals, such as MAPK/ERK, to modulate inflammatory and immune responses [20–25]. DENV infection can induce MIF secretion [26, 27], and the concentration of MIF is positively correlated with dengue severity [28]. Furthermore, DENV infection-induced disease was found to be less severe in MIF knockout (*Mif*^*-/-*^) mice than in normal mice [29]. However, it is unclear whether MIF is directly involved in NS1-induced glycocalyx degradation. To address this question, we studied the effects of NS1 on the secretion of MIF, HPA-1, MMP-9, and CD138 both *in vitro* and *in vivo*. We found that the levels of MIF, HPA-1, MMP-9, and CD138 were all increased in the serum of dengue patients. Similar results were found both *in vitro* and *in vivo* after recombinant NS1 challenge. Most importantly, the NS1-induced increases in HPA-1, MMP-9, and CD138 were all inhibited in the presence of MIF inhibitors or antibodies both *in vitro* and *in vivo*, indicating that NS1-induced MIF secretion may play an important role in the pathogenesis of DENV NS1-induced glycocalyx degradation and vascular leakage.

## Results

### Comparison of NS1, HPA-1, MMP-9, CD138 and MIF serum concentrations in dengue patients

**Fig 1:**
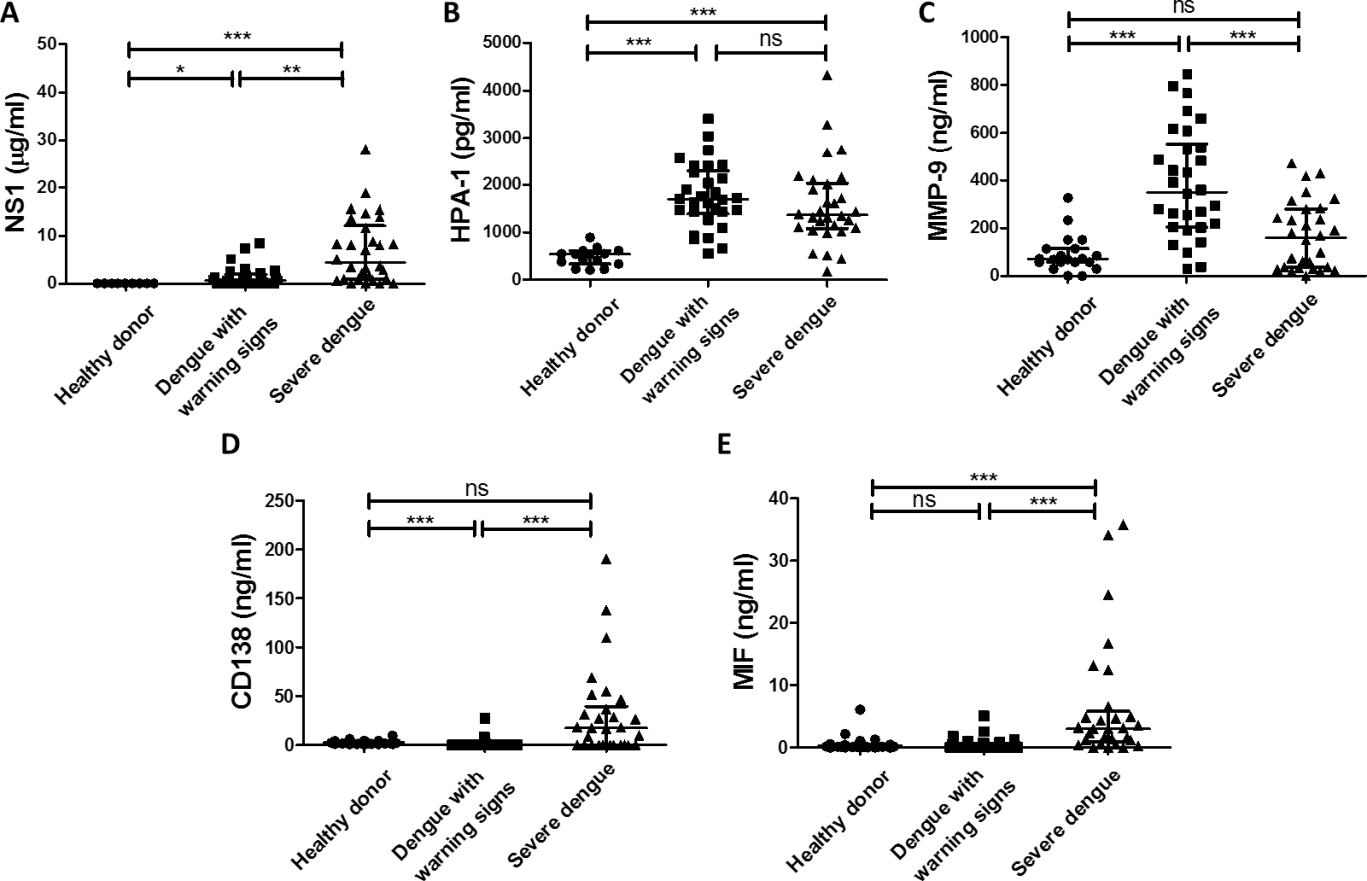
The serum concentrations of NS1, HPA-1, MMP-9, CD138 and MIF in healthy donors and dengue patients. The serum concentrations of **(A)** NS1 **(B)** HPA-1, **(C)** MMP-9, **(D)** CD138 and **(E)** MIF in healthy donors and dengue patients were compared as indicated. *P<0.05; **P<0.01; ***P<0.001; ns: not significant; ANOVA with Dunn's test for multiple comparisons (panel A, D,and E), Tukey's multiple comparisons test (panel B and C).

**Fig 2:**
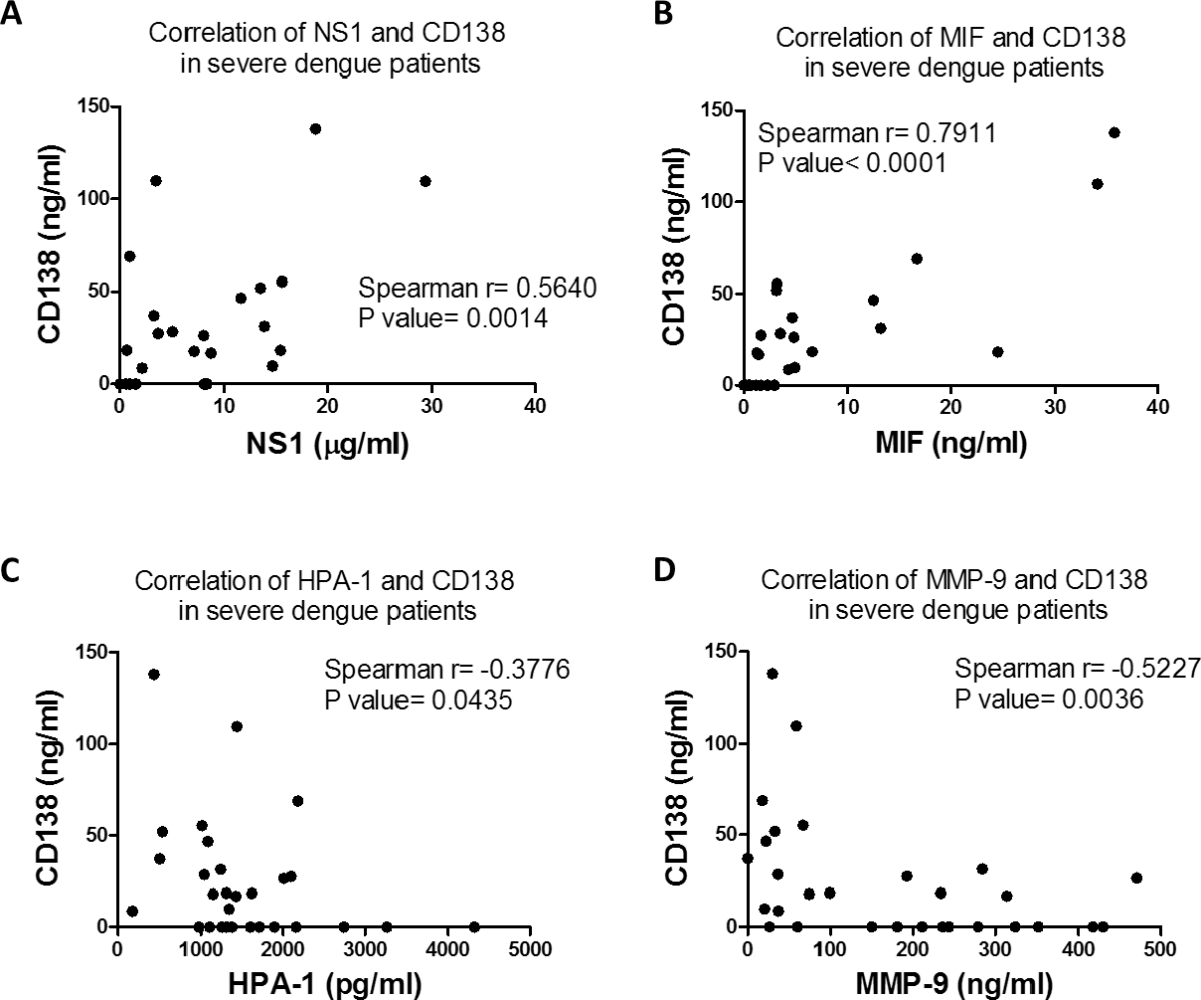
The correlations of serum NS1, MIF, HPA-1, MMP-9 and CD138 levels in severe dengue patients. The correlations of the concentrations of **(A)** NS1, **(B)** MIF, **(C)** HPA-1 and **(D)** MMP-9 and CD138 in the same group of severe dengue patients were plotted. Linear regressions were analyzed using nonparametric correlation test (panel A, B, C and D).

The concentrations of NS1 and glycocalyx degradation-related molecules in serum samples from healthy donors and dengue patients were measured by ELISA. The concentrations of NS1 and HPA-1 were increased in both dengue patients with warning signs and severe dengue patients (Fig 1A and 1B). The concentrations of MMP-9 were also significantly elevated in dengue patients with warning signs but not in severe dengue patients (Fig 1C). The concentrations of CD138 and MIF were significantly elevated in the serum of severe dengue patients compared to dengue patients with warning signs (Fig 1D and 1E). To further elucidate the correlation between CD138 and the other molecules, the serum concentrations of NS1, MIF, HPA-1 and MMP-9 in severe dengue patients were plotted against the concentration of CD138 (Fig 2). Only NS1 and MIF showed a positive correlation with CD138 in the sera of severe dengue patients (Fig 2A and 2B). Additionally, the viral load of severe dengue patients did not show a significantly positive correlation with any factor mentioned above (S1 Fig). These results suggest that NS1 and MIF may play important roles in CD138 shedding in severe dengue patients.

### DENV NS1-induced endothelial HPA-1 secretion leads to glycocalyx degradation and hyperpermeability and is MIF dependent

**Fig 3:**
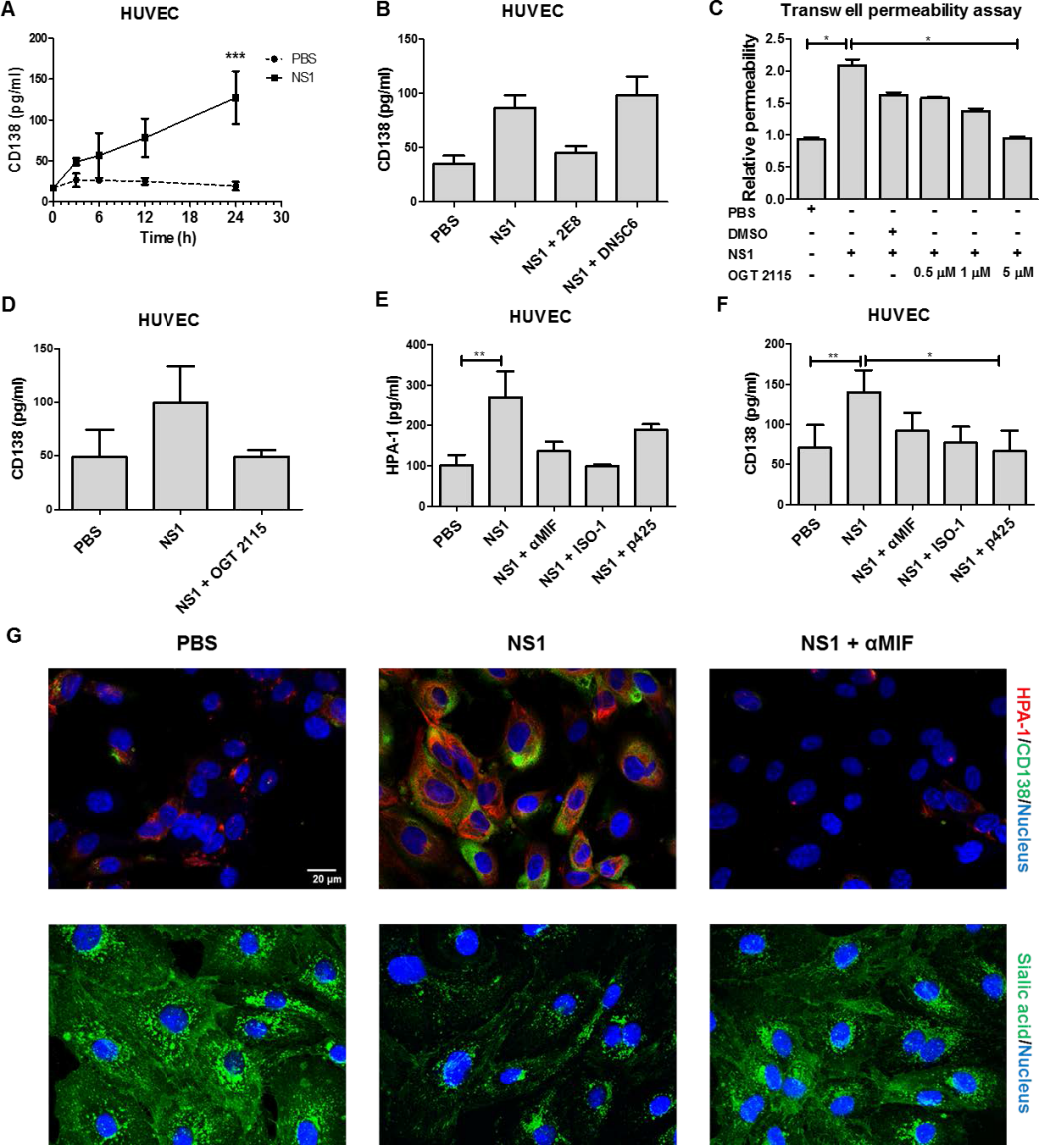
DENV NS1-induced HPA-1 secretion leading to glycocalyx degradation and hyperpermeability is MIF dependent. **(A)** HUVECs were treated with PBS or 20 μg/ml NS1 recombinant proteins for the indicated time, followed by collection of the supernatants for CD138 detection by ELISA. (n = 4) **(B)** HUVECs were treated with PBS, 20 μg/ml NS1, or 20 μg/ml NS1 mixed with 10 μg/ml anti-NS1 antibodies (2E8 or DN5C6). After 24 h of incubation, the culture medium was collected, and the concentration of CD138 was determined by ELISA. (n = 3) **(C)** HUVECs seeded as monolayers in upper Transwell chambers were treated with PBS, 20 μg/ml NS1 or 20 μg/ml NS1 mixed with DMSO or the indicated concentration of OGT 2115. After 24 h, endothelial permeability was determined by a Transwell permeability assay, as described in the Materials and Methods section. (n = 3) **(D)** HUVECs were treated with PBS or 20 μg/ml NS1 with or without 5 μM OGT 2115. After 24 h, the cell culture medium was collected, and the CD138 concentration was measured by ELISA. (n = 5) **(E)** HUVECs were treated with PBS or 20 μg/ml NS1 with or without 100 μM p425, 50 μM ISO-1, or 10 μg/ml anti-MIF antibodies, as indicated. After 24 h, the cell culture medium was collected, and the HPA-1 concentration was measured by ELISA. (n = 3) **(F)** HUVECs were treated with PBS or 20 μg/ml NS1 with or without p425, ISO-1 or anti-MIF antibodies. After 24 h, the cell culture medium was collected, and the CD138 concentration was measured by ELISA. (n = 5) **(G)** HUVECs were treated with PBS or NS1 (20 μg/ml) with or without anti-MIF polyclonal antibodies (10 μg/ml) for 24 h. The distribution of HPA-1 (red) and CD138 (green) was assessed by staining with specific antibodies. Sialic acid expression on HUVECs monolayers was assessed by staining with WGA-FITC (green). *P<0.05, **P<0.01, ***P<0.001; ns, not significant; unpaired t-test (panel A), Kruskal-Wallis ANOVA (panel B, C, D, E and F).

According to a previous study, NS1 can induce endothelial cells to secrete HPA-1 to disrupt the endothelial glycocalyx, and this disruption is characterized by CD138 shedding [18]. To further investigate the underlying mechanism of NS1-induced HPA-1 secretion, human umbilical vein endothelial cells (HUVECs) were stimulated with NS1 for various durations. The results show that CD138 was significantly increased in cell culture medium after 24 h of NS1 treatment (Fig 3A). To confirm that this effect was induced by NS1, anti-NS1 monoclonal antibody (mAb) was used to block the effect of NS1. Anti-NS1 mAb 2E8, which can inhibit NS1-induced vascular leakage, was able to inhibit NS1-induced CD138 shedding (Fig 3B) [19]. In contrast, another anti-NS1 mAb (DN5C6), which was used as a negative control, failed to inhibit NS1-induced CD138 shedding from endothelial cells (Fig 3B) [19]. NS1 stimulation also increased the active HPA-1 level in endothelial cell lysates, which was abolished by mAb 2E8 but not control mouse IgG (S2A Fig). To confirm that HPA-1 is involved in NS1-induced endothelial hyperpermeability and CD138 shedding, recombinant HPA-1 protein and the HPA-1 inhibitor OGT 2115 were used. Inoculating the mice with native but not heat-denatured recombinant HPA-1 directly induced vascular leakage (S2B Fig). Furthermore, cotreatment with OGT 2115 attenuated NS1-induced endothelial hyperpermeability (Fig 3C) and reduced CD138 release to levels similar to those of the phosphate-buffered saline (PBS) control *in vitro* (Fig 3D).

In addition to HPA-1, MIF is also capable of inducing endothelial hyperpermeability [19]. As a result, the MIF concentration in the conditioned medium obtained from NS1-stimulated HUVECs was measured. The result shows that 10 μg/ml NS1 was sufficient to induce MIF secretion (S3A Fig). Furthermore, the conditioned medium obtained from NS1-stimulated HUVECs could induce endothelial hyperpermeability and CD138 shedding after incubation with another HUVEC monolayer (S3B and S3C Fig). To clarify which protein mediates NS1-induced endothelial hyperpermeability and CD138 shedding, MIF-blocking antibodies, the HPA-1 inhibitor OGT 2115 and NS1-blocking antibodies were used. The results show that both the anti-MIF antibodies and OGT 2115 attenuated NS1-stimulated conditioned medium-induced HUVEC hyperpermeability and CD138 shedding (S3D and S3E Fig). Interestingly, the NS1-blocking antibody 2E8 only partially diminished the conditioned medium-induced HUVEC hyperpermeability but not the conditioned medium-induced HUVEC CD138 shedding (S3D and S3E Fig). Cotreatment with MIF inhibitors (anti-MIF antibodies, ISO-1, and p425) and NS1 also attenuated the NS1-induced HPA-1 secretion (Fig 3E) and CD138 shedding of endothelial cells (Fig 3F). In addition, we also visualized the HPA-1 expression, CD138 deposition and sialic acid expression using immunofluorescence with anti-HPA antibodies, anti-CD138 antibodies and wheat germ agglutinin (WGA) lectin which can bind to sialic acids and other sugars such as *N*-acetylglucosamine. As shown in Fig 3G, NS1-induced HPA-1 expression, CD138 deposition and sialic acid degradation could also be rescued by MIF inhibition. However, the HPA-1 inhibitor OGT 2115 failed to affect MIF secretion, suggested that MIF is the upstream effector of HPA-1 (S3F Fig). To further clarify this hypothesis, recombinant MIF was used. The results show that MIF increased CD138 shedding and the active HPA-1 level in HUVECs (S4A and S4B Fig). The increased HPA-1 expression and CD138 deposition after MIF stimulation could also be observed by immunofluorescence (S4C Fig). These results indicate that NS1 can induce the MIF-mediated secretion of active HPA-1, leading to endothelial glycocalyx degradation.

### DENV NS1 induces MMP-9 secretion in THP-1 cells and leukocytes

**Fig 4:**
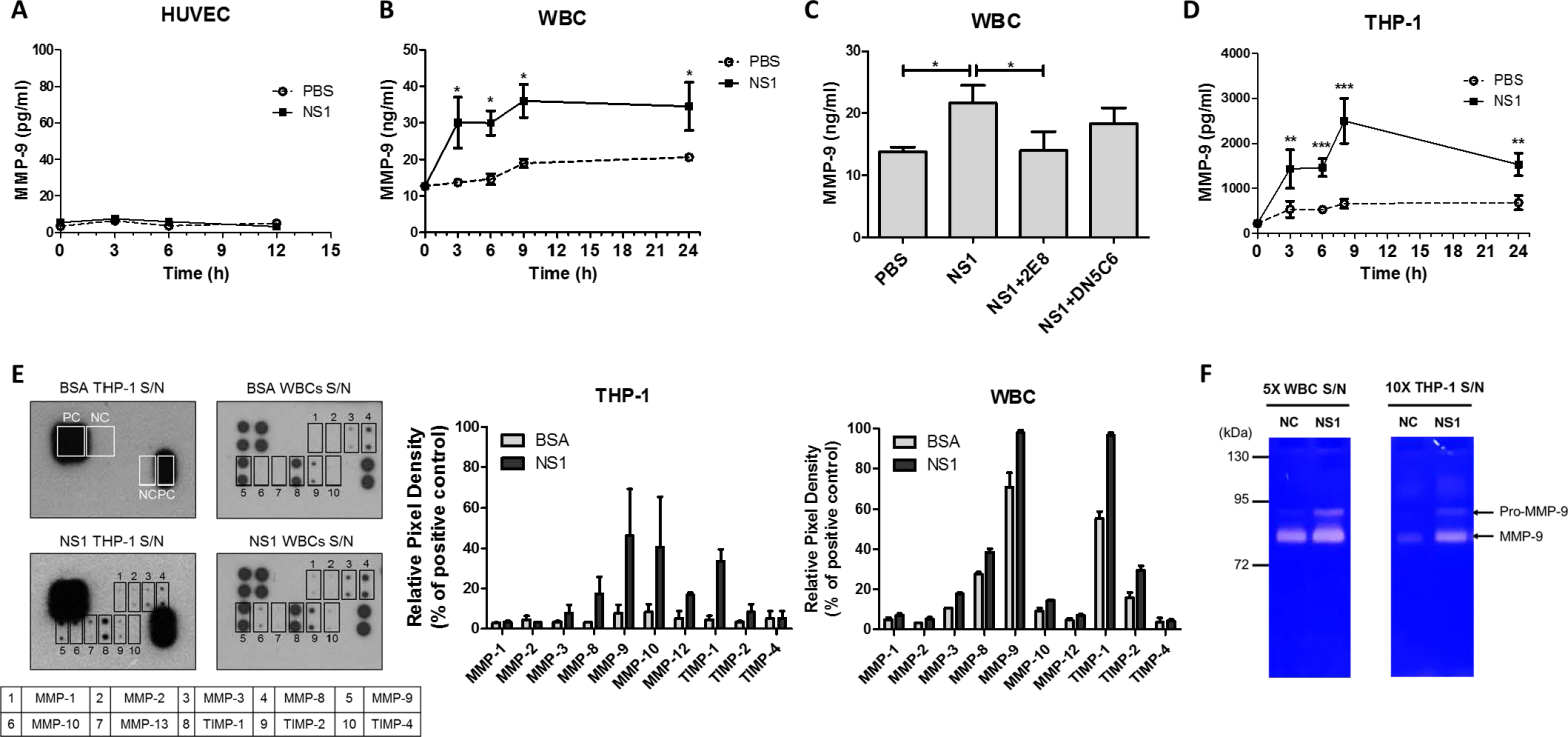
NS1 induces MMP-9 secretion from human leukocytes but not HUVECs. **(A)** HUVECs were treated with PBS or NS1 for the indicated times; then, the supernatants were collected for MMP-9 detection by ELISA. (n = 3) **(B)** Isolated human leukocytes (WBCs) and **(D)** PMA-activated THP-1 cells were treated with PBS or NS1, and the culture medium was collected at the indicated times. The concentration of MMP-9 in the culture medium was determined by ELISA. (n = 4) **(C)** Isolated WBCs were treated with PBS, NS1, or NS1 mixed with anti-NS1 antibodies for 24 h, and the concentration of MMP-9 in the culture medium was determined by ELISA. (n = 4) **(E)** After PMA activation, THP-1 and primary isolated WBCs were subjected to the desired treatment. After 24 h, the cell culture supernatants were collected. An MMP antibody array that detects various MMPs and TIMPs was used to assess the major subclass of MMPs induced by NS1. THP-1 cells or WBCs were stimulated with PBS or NS1; then, the culture supernatants were collected and analyzed for extracellular matrix proteins. Membranes of the human MMP antibody array were probed with the supernatant collected from bovine serum albumin (BSA)-treated THP-1 cells, BSA-treated WBCs, 20 μg/ml NS1-treated THP-1 cells or NS1-treated WBCs. The quantification of MMPs array membranes was analyzed by ImageJ. PC, positive control; NC, negative control. **(F)** After treatment with 20 μg/ml NS1 for 24 h, the 5X-concentrated WBCs and 10X-concentrated THP-1 supernatants were analyzed by electrophoresis with a 7.5% acrylamide gel containing gelatin. The gel was stained with Coomassie blue to reveal the white bands corresponding to the proteolysis of gelatin by MMPs. S/N, supernatant; *P<0.05, **P<0.01, ***P<0.001; unpaired t-test (panel B and D), Kruskal-Wallis ANOVA (panel C).

Since MMPs can degrade the endothelial glycocalyx and several MMPs are upregulated during DENV infection [30, 31], we speculated that MMPs are involved in NS1-induced glycocalyx degradation. Because a previous study has indicated that an increase in circulating MMP-9 levels is associated with dengue disease severity [32], we first examined whether NS1 induces MMP-9 secretion. However, we found that NS1 barely induced MMP-9 secretion in HUVECs (Fig 4A). Since MMPs are primarily secreted by leukocytes (white blood cells, WBCs), including neutrophils and monocytes [33], we tested whether NS1 could induce MMP secretion in human leukocytes, peripheral blood mononuclear cells (PBMCs), and THP-1 human monocytes. We found that NS1 induced MMP-9 secretion in freshly isolated leukocytes after 3 h of stimulation (Fig 4B), an effect that was attenuated by the NS1-blocking antibody 2E8 (Fig 4C). Similarly, NS1 induced phorbol myristate acetate (PMA)-activated THP-1 cells to secrete MMP-9 after incubation for 3 h (Fig 4D). However, neither MIF nor MMP-9 secretion was significantly induced in NS1-stimulated PBMCs compared to the controls (S5A and S5B Fig). To obtain the secretion profile of MMPs, we used an MMP antibody array to analyze which MMPs were increased by NS1 in PMA-activated THP-1 cells and leukocytes. The results show that MMP-8, MMP-9, and TIMP-1 were increased in the culture medium of NS1-treated THP-1 cells and leukocytes (Fig 4E). To confirm the activity of MMP-9, cell culture medium from NS1-treated PMA-activated THP-1 cells and leukocytes were analyzed using a gelatin zymography assay, which showed that NS1 induced both THP-1 cells and leukocytes to secrete pro-MMP-9 and activated MMP-9 (Fig 4F).

### DENV NS1-induced MMP-9 secretion from THP-1 cells increases endothelial permeability and glycocalyx degradation

**Fig 5:**
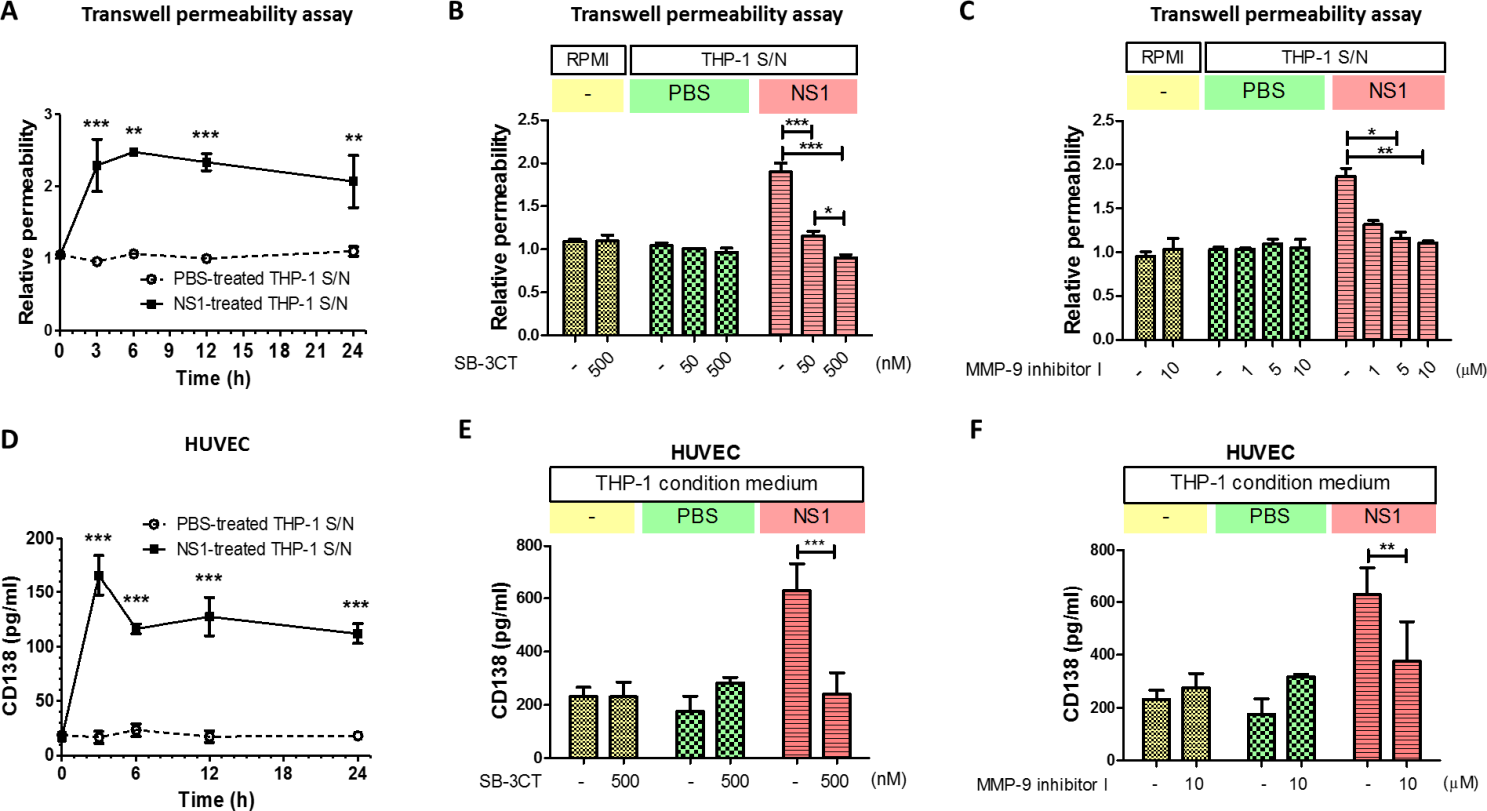
DENV NS1-induced MMP-9 secretion from THP-1 cells causes endothelial hyperpermeability and glycocalyx degradation. **(A)** PMA-activated THP-1 cells were incubated with NS1; the supernatants were collected at the indicated times and incubated with HUVECs for 24 h. The endothelial permeability was determined by a Transwell permeability assay (n = 3), as described in the Materials and Methods section. **(B) (C)** PMA-activated THP-1 cells were incubated with NS1 for 24 h, and the resulting supernatants were collected. Fresh RPMI 1640 or NS1-treated THP-1 cell culture supernatants were incubated with HUVECs, with or without the indicated concentrations of **(B)** SB-3CT or **(C)** MMP-9 inhibitor I. After 24 h of incubation, endothelial permeability was determined by Transwell permeability assay. (n = 3) **(D)** NS1-treated THP-1 cell-conditioned medium was collected at the indicated times and incubated with HUVECs for 24 h. The concentration of CD138 in the supernatant was determined by ELISA. (n = 3) **(E) (F)** Fresh RPMI 1640 or NS1-treated THP-1 cell culture supernatant was mixed with or without the indicated concentrations of **(E)** SB-3CT or **(F)** MMP-9 inhibitor I and incubated with HUVECs for 24 h. The concentration of CD138 in the supernatant was determined by ELISA (n = 3). S/N, supernatant; **P<0.01, ***P<0.001; unpaired t-test (panel A and D), Kruskal-Wallis ANOVA (panel B, C, E and F).

To test whether the NS1-induced MMP-9 secretion of THP-1 cells causes endothelial hyperpermeability, the supernatant from NS1-treated THP-1 cells was incubated with HUVECs, and both permeability and CD138 shedding were examined. The results show that after 3 h of treatment, the supernatant from NS1-treated THP-1 cells increased endothelial permeability (Fig 5A). This phenomenon was attenuated in the presence of the MMP-2/MMP-9 inhibitor SB-3CT and the MMP-9-specific inhibitor MMP-9 inhibitor I (Fig 5B and 5C). The supernatant from untreated or PBS-treated THP-1 cells did not alter endothelial permeability (Fig 5B and 5C). Similarly, the supernatant from NS1-treated THP-1 cells also induced CD138 shedding from HUVECs (Fig 5D), and this effect was diminished by SB-3CT and MMP-9 inhibitor I (Fig 5E and 5F). Similar results were found for the supernatant obtained from NS1-stimulated leukocytes (S6 Fig). The NS1-blocking antibody 2E8 was used to block NS1 remaining in the supernatant, and it did not alter the endothelial permeability induced by the supernatant, showing that the effect of NS1 remaining in the supernatant is negligible (S6 Fig). These results indicate that NS1 can induce MMP-9 secretion in leukocytes, leading to endothelial barrier dysfunction.

### MIF is required for DENV NS1-induced MMP-9 secretion

As MIF is a crucial mediator of NS1-induced vascular leakage and an upstream regulator of MMP-9 [19, 26, 34, 35], we tested whether NS1-induced MMP-9 secretion in leukocytes is also MIF dependent. First, we wanted to confirm whether NS1 induces the secretion of MIF from leukocytes and THP-1 cells. Since a previous study has shown that NS1 increases the expression of IL-6 and IL-8 in PBMCs [16], we also measured the concentrations of IL-6 and IL-8 after NS1 stimulation. The secretion of MIF from NS1-stimulated leukocytes steadily accumulated up to 2000 pg/ml (S7A Fig). NS1 could also enhance the secretion of IL-6 and IL-8 from leukocytes, but the concentrations dropped rapidly after 3 h (S7 B and S7C Fig). However, in THP-1 cells, NS1 only enhanced the secretion of MIF, not IL-6 or IL-8 (S7D–S7F Fig). These results suggest that MIF is the major cytokine induced by the DENV NS1 stimulation of leukocytes and monocytes.

**Fig 6:**
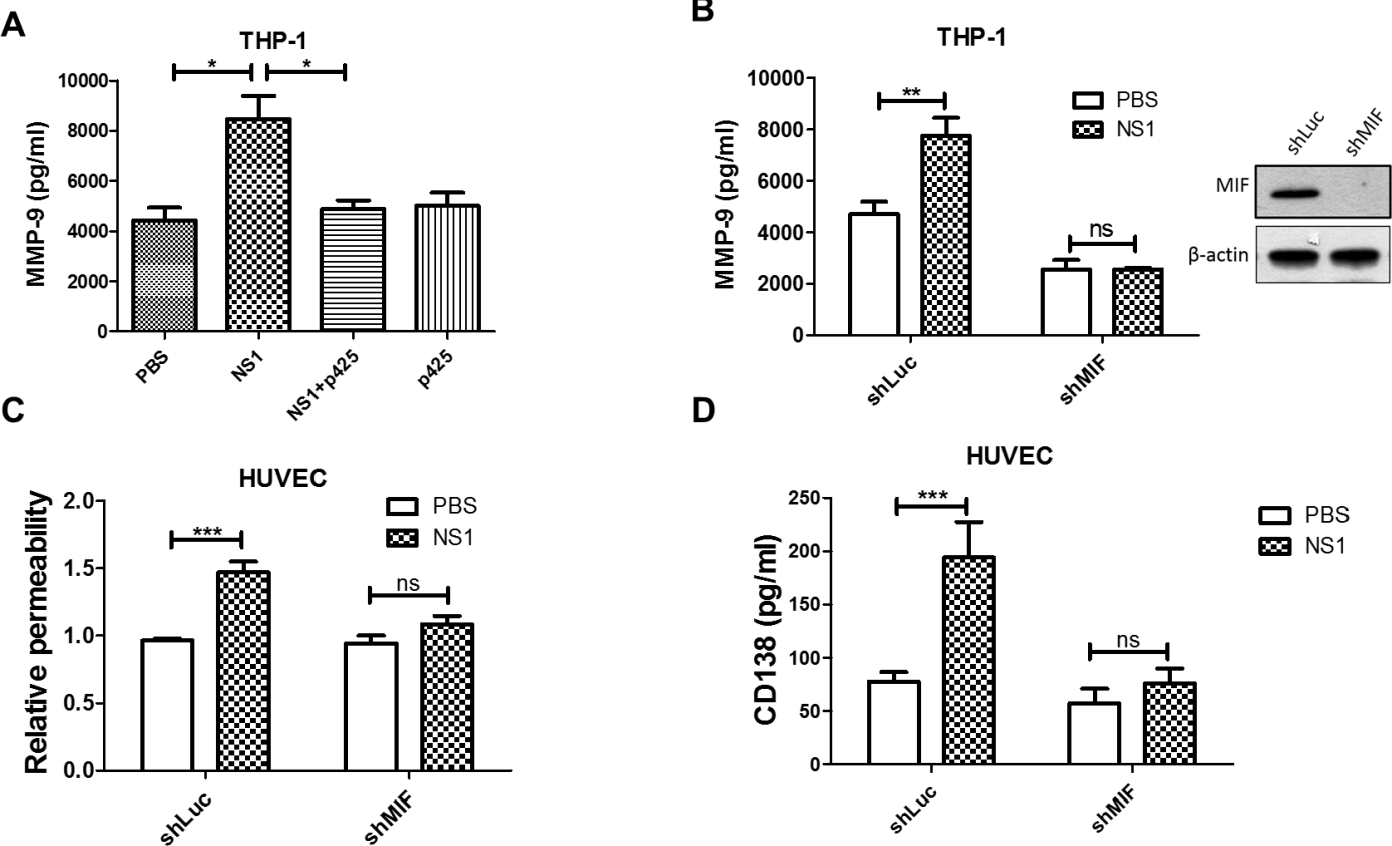
NS1-induced MMP-9 secretion from THP-1 cells is regulated by MIF. **(A)** PMA-activated THP-1 cells were treated with PBS, NS1, or NS1 with p425 for 24 h, and the concentration of MMP-9 in the supernatant was determined by ELISA (n = 5). **(B)** The MMP-9 levels in the supernatants of PMA-activated THP-1-shLuc and THP-1-shMIF cell cultures were detected by ELISA after incubation with or without NS1 for 24 h. (n = 3) **(C)** PMA-activated THP-1-shLuc and THP-1-shMIF cells were incubated with PBS or NS1 for 24 h; then, the supernatant was collected and incubated with HUVEC monolayers grown in upper Transwell chambers. After 24 h of incubation, endothelial permeability was determined using streptavidin-HRP and TMB. (n = 6) **(D)** The supernatant of THP-1-stimulated HUVEC cultures was collected, and the CD138 concentration was determined by ELISA (n = 5). *P<0.05, **P<0.01, ***P<0.001; ns, not significant; Kruskal-Wallis ANOVA (panel A), unpaired t-test (panel B, C and D).

To clarify whether NS1-induced MMP-9 secretion is mediated by MIF, the MIF inhibitor p425 and MIF short-hairpin RNA (shRNA) were used. The ELISA results show that inhibiting MIF with its inhibitor p425 abolished NS1-induced MMP-9 secretion, while p425 alone did not affect MMP-9 secretion (Fig 6A). Next, we used shRNA to knockdown MIF expression in THP-1 cells. Western blot analysis showed that the expression of MIF was diminished by shMIF compared to the shLuc scrambled control (Fig 6B). Furthermore, the knockdown of MIF decreased NS1-induced MMP-9 secretion from THP-1 cells (Fig 6B), and the culture supernatant from shMIF THP-1 cells failed to increase endothelial permeability or CD138 shedding (Fig 6C and 6D). We also knocked down MIF expression in HUVECs and measured the permeability under NS1 stimulation as a comparison. Consistent with our previous study, the knockdown of MIF in HUVECs diminished NS1-induced endothelial hyperpermeability (S8A and S8B Fig). These results suggest that MIF acts on both endothelial cells and leukocytes to mediate NS1-induced endothelial hyperpermeability.

### DENV NS1 induces MIF, HPA-1, MMP-9 and CD138 secretion in mice

**Fig 7:**
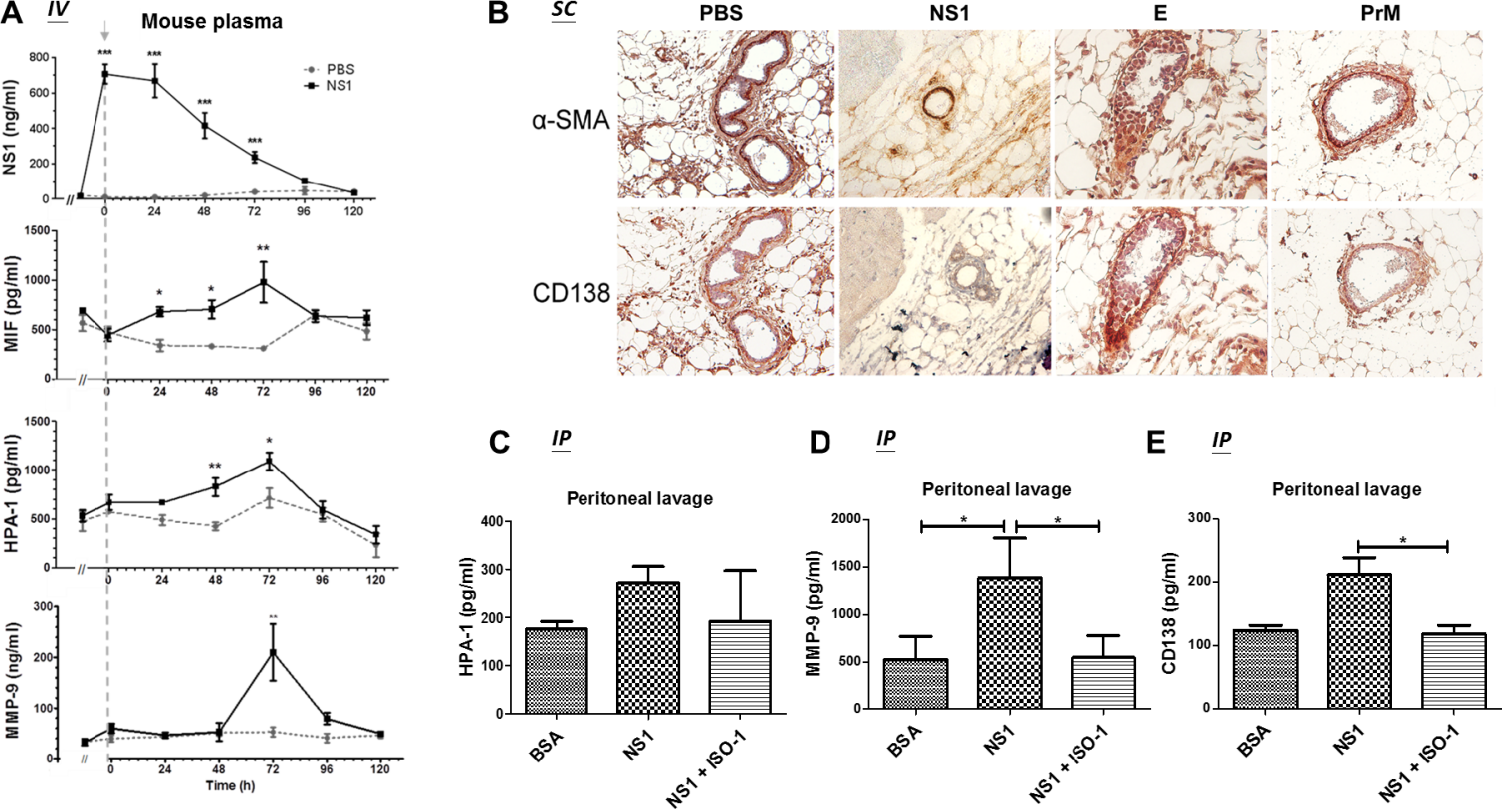
MIF inhibition attenuates DENV NS1-induced glycocalyx degradation in mice. **(A)** Before the injection of PBS or NS1, the blood of 8- to 12-week-old BALB/c mice (n = 3) was collected by orbital sinus sampling with 10% citrate. Next, the mice were intravenously injected with 50 μg of NS1 or 100 μl of PBS. Blood samples were collected from the mice immediately after injection and every 24 h thereafter until 120 h. The plasma concentrations of NS1, MIF, HPA-1, and MMP-9 were measured by ELISA. Arrow, injection time point. **(B)** BALB/c mice received two subcutaneous injections of PBS, NS1, or recombinant E or prM at the same location within 24 h. One day after the second injection, the mice were sacrificed, and a series of skin tissue sections were hybridized with anti-α-SMA and anti-CD138 antibodies and stained with DAB (brown). **(C-E)** BALB/c mice (n = 5) were intraperitoneally injected with BSA or NS1 with or without an MIF inhibitor (ISO-1), and the peritoneal lavage fluid was collected 24 h after the injection. The concentrations of **(C)** HPA-1, **(D)** MMP-9 and **(E)** CD138 in the peritoneal lavage fluid were measured by ELISA. *P<0.05, **P<0.01, ***P<0.001; unpaired t-test (panel A), Kruskal-Wallis ANOVA (panel C, D and E).

To further confirm that NS1 can induce MIF, HPA-1, MMP-9 and CD138 secretion *in vivo*, we injected 50 μg of NS1 into the tail veins of mice, and blood samples were collected every 24 h. The concentrations of NS1, MIF, HPA-1, and MMP-9 were measured by ELISA. The results show that the peak concentration of NS1 in the plasma of mice after injection was approximately 0.75 μg/ml, which falls in the range of NS1 circulating in the bloodstream of DENV-infected patients, estimated as 0.01–50 μg/ml [36]. The concentration of circulating NS1 in mice gradually decreased after the injection and was cleared from the plasma after 96 h (Fig 7A). The MIF concentration increased 24 h after the injection, peaked at 72 h, and then dropped to basal levels after 96 h (Fig 7A). The upregulation of HPA-1 occurred later than that of MIF, as it was significantly elevated after 48 h, but it also peaked at 72 h and then dropped to basal levels after 96 h (Fig 7A). The secretion of MMP-9 did not increase until 72 h, and then it returned to basal levels at 96 h (Fig 7A), exhibiting an increase over a relatively short period.

### MIF inhibition attenuates DENV NS1-induced endothelial glycocalyx degradation in mice

To further investigate whether NS1 causes endothelial glycocalyx degradation in mice, the skin tissues of mice after two sequential subcutaneous injections of NS1 were fixed for immunohistochemical (IHC) staining. Costaining with the endothelial marker α-SMA revealed CD138 only in the samples with two injections of PBS, E or prM (Fig 7B). After two sequential injections of NS1, endothelial cells lost their CD138 staining (Fig 7B). In addition, the intraperitoneal injection of NS1 significantly induced HPA-1, MMP-9, and CD138 secretion, and coinjection of ISO-1 significantly abolished the secretion of MMP-9 and CD138 but not HPA-1 found by the peritoneal lavage (Fig 7C–7E). Furthermore, the inhibition of MIF and MMP-9 also attenuated NS1-induced vascular leakage in mice (S9 Fig). These results suggest that MMP-9 induced by NS1-stimulated leukocytes may play an important role in endothelial glycocalyx degradation.

## Discussion

**Fig 8:**
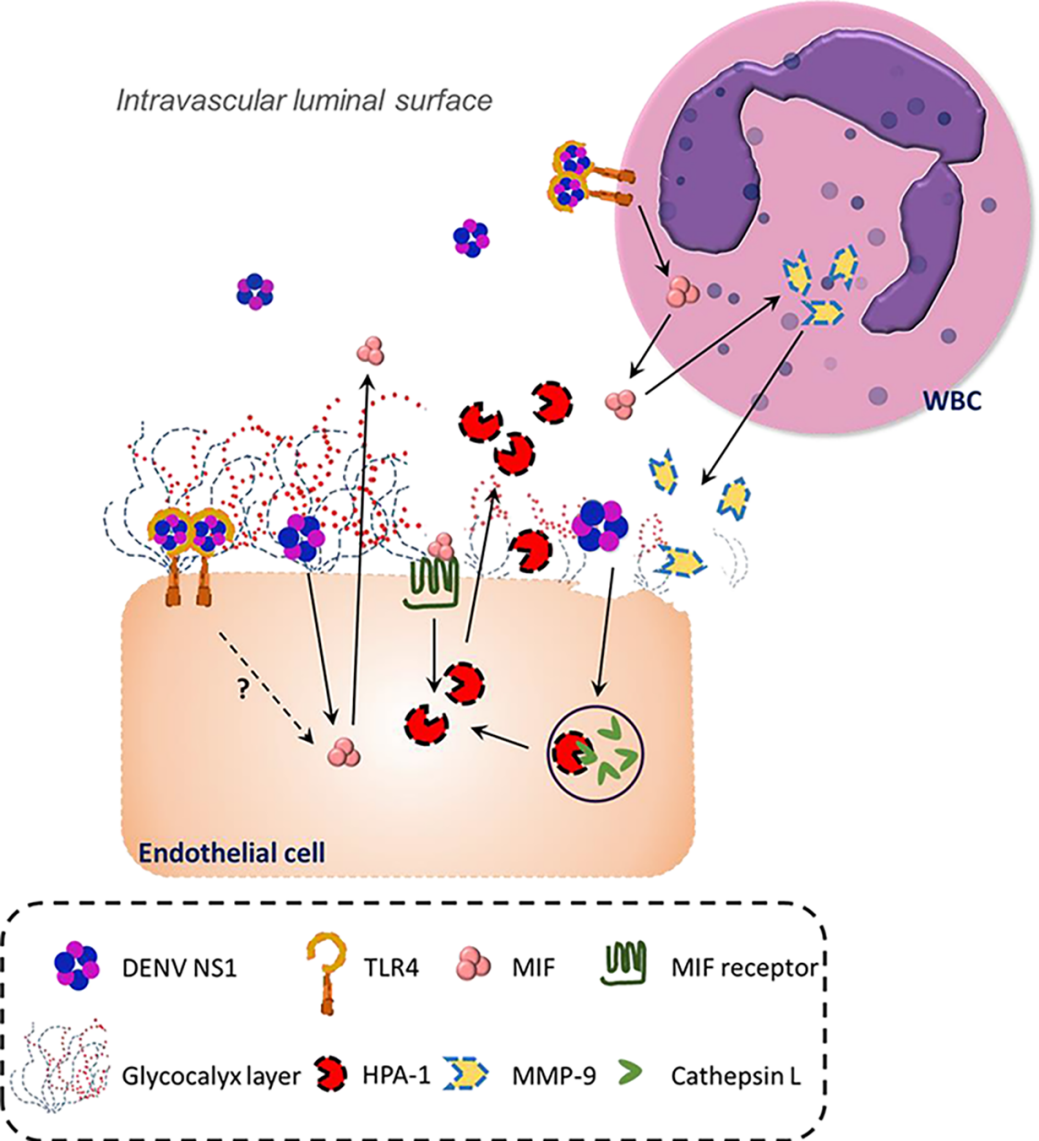
The proposed mechanisms of DENV NS1-induced endothelial glycocalyx degradation. Circulating DENV NS1 may bind to endothelial cells via TLR4 or other molecules to stimulate the secretion of MIF, which in turn elevates the protein level of active HPA-1, causing it to digest glycocalyx on the intravascular luminal surface of endothelial cells. On the other hand, NS1 can also bind to TLR4 on leukocytes to secrete MMP-9, causing glycocalyx degradation. Even though the effector molecules causing glycocalyx degradation (HPA-1 and MMP-9) are different, MIF is part of both HPA-1 secretion by endothelial cells and MMP-9 secretion by leukocytes.

In this study, we first observed that the concentrations of NS1, MIF, HPA-1, MMP-9 and CD138 in the serum of dengue patients were increased. However, only the concentrations of NS1 and MIF showed a positive correlation with CD138 in severe dengue patients. Next, we showed that the DENV NS1 stimulation of endothelial cells and leukocytes could induce HPA-1 and MMP-9 secretion, respectively, causing endothelial glycocalyx degradation and hyperpermeability. Most importantly, both *in vitro* and *in vivo* data showed that dengue NS1-induced HPA-1 and MMP-9 secretion was MIF dependent. Therefore, these results suggest that MIF is a central modulator of both direct and indirect dengue NS1-induced endothelial glycocalyx degradation (Fig 8).

Previously, Puerta-Guardo *et al*. showed that HPA-1 is involved in NS1-induced glycocalyx degradation and hyperpermeability [18]. However, MMPs were not discussed in the mechanism, even though they are the main enzymes that degrade endothelial glycocalyx [11, 12]. It is known that DENV infection induces dendritic cells to secrete MMP-9 [31]. In this study, we further demonstrated that the NS1 stimulation of leukocytes but not endothelial cells nor PBMCs could induce MMP-9 secretion. It is known that DENV NS1 can induce neutrophil extracellular traps, which results in the release of tertiary granules containing MMP-9 [37, 38]. A previous study has also shown that MIF can mediate the secretion of MMP-9 from neutrophils [39]. Since neutrophils are a major population of leukocytes, taken together, these results suggest that NS1-stimulated neutrophils may represent the main contributors to MMP-9 secretion in the blood. Therefore, even though neutrophils are not the primary target of DENV infection [40, 41], the secretion of MMP-9 from neutrophils induced by NS1 may also contribute to vascular leakage during DENV infection.

Interestingly, although it has been shown in previous studies that the concentrations of MMPs are increased in dengue patients [13, 32] and MMP-9 upregulation is positively correlated with the disease severity and vascular leakage of dengue [31, 32, 42, 43], we observed a significant increase in the serum level of MMP-9 only in dengue patients with warning signs, not in severe dengue patients. From the *in vivo* mouse study, we noticed that the secretion of MMP-9 occurred within a smaller time window than that of HPA-1 in mice after NS1 challenge. Therefore, it is possible that the discrepancy in the MMP-9 level in dengue patients between this and previous studies may be due to differences in the timing of sample collection. Because the specific day post-onset of symptoms that samples were collected was not available in the records of our dengue patients, we could not exclude the possibility of variation arising from different sampling times. Further study monitoring the sequential changes in the serum levels of MMP-9 and other glycocalyx-related molecules along with disease development is required to clarify their roles in dengue pathogenesis.

From the results of the MMP antibody array, we also found that MMP-8 and tissue inhibitor of metalloproteinases 1 (TIMP-1) were upregulated by NS1-stimulated leukocytes. TIMP-1, which is a potent inhibitor of MMPs, can form a complex with pro-MMP-9 at a 1:1 stoichiometric relationship to inhibit its activation [44, 45]. However, neutrophil elastase can inactivate TIMP-1 in the complex to free pro-MMP-9, such that it can be activated by MMP-3 [46]. In addition, myeloperoxidase, which is most abundantly expressed by neutrophils, can also inactivate TIMP-1 via generating hypochlorous acid [47]. These possible mechanisms may explain why MMP-9 activity was not abrogated in the presence of TIMP-1 in NS1-stimulated leukocytes.

A previous study has shown that NS1 can induce PBMCs to secrete IL-6 and IL-8 via Toll-like receptor 4 (TLR4), leading to vascular leakage [16]. However, in this study, we found that the secretion of IL-6 and IL-8 dropped rapidly after 3 h of NS1-stimulation in leukocytes (S7B and S7C Fig). In contrast, MIF steadily accumulated in the supernatant of leukocyte cultures after NS1 treatment, and the concentration of MIF was higher than that of IL-6 and IL-8 (S7 Fig). As NS1 needs at least 24 h to induce endothelial glycocalyx degradation (Fig 3A), we speculated that IL-6 and IL-8 are not very involved in NS1-induced endothelial glycocalyx degradation. This speculation is consistent with a recent study performed by Glasner *et al*., which found that DENV NS1 does not induce HMEC-1 human endothelial cells to secrete TNF-α, IL-6 or IL-8 and that blocking these cytokines does not affect DENV NS1-induced endothelial hyperpermeability [48]. On the other hand, the same study found that inhibition of HPA-1 prevents DENV NS1-induced endothelial hyperpermeability [48]; however, MIF was not measured. In our previous study and in this study, we demonstrated that NS1 induced HMEC-1 cells or HUMECs to secrete MIF, causing endothelial hyperpermeability [19]. In addition, we further demonstrated that both the secretion of HPA-1 and the shedding of CD138 induced by NS1-stimulation of endothelial cells are mediated by MIF.

Due to MIF regulating the secretion of both MMP-9 and HPA-1 and because CD138 shedding was also directly affected by MIF signaling, MIF may be an upstream regulator of DENV NS1-induced glycocalyx degradation. However, the mechanism of how MIF causes HPA-1 and MMP-9 secretion is still unclear. A previous study has shown that MIF induces MMP-9 expression in macrophages via the MAPK pathway [35]. MIF is also known to activate NF-κB signaling through binding to CD74 [49]. Additionally, HPA-1 mRNA expression is elevated in an NF-κB-dependent manner during hypoxia [50]. Therefore, it is possible that MIF contributes to the secretion of HPA-1 and MMP-9 via the MAPK/NF-κB pathway. However, from our *in vivo* study, we also noticed that NS1-induced MMP-9 secretion and CD138 shedding were significantly attenuated by MIF inhibition, whereas the attenuation of HPA-1 secretion was not as significant. Serum samples from severe dengue patients also showed no linear relationship between the concentrations of MIF and HPA-1 (S10 Fig). These results may suggest that in addition to MIF, other factors may participate in the regulation of HPA-1 secretion *in vivo*.

Taken together, our results suggest that NS1 may contribute to vascular leakage through different mechanisms during DENV infection. DENV NS1 may bind to the TLR4 of leukocytes, inducing the secretion of cytokines and MMPs, or it may directly bind to endothelial cells, inducing the secretion of HPA-1, both of which can cause glycocalyx degradation and subsequent vascular leakage. Consequently, NS1 may represent an important viral factor that causes vascular leakage and glycocalyx degradation during DENV infection. Indeed, antibodies against NS1 have been shown to be protective against DENV infection in mice [17, 51, 52]. Furthermore, MIF may represent the primary host factor that mediates NS1-induced glycocalyx degradation. Studies focusing on the development of neutralizing antibodies or small molecules against MIF may facilitate the development of drugs to prevent or treat severe dengue [53].

## Materials and methods

### Experimental design

The aim of this study was to clarify the mechanism of DENV infection-induced endothelial glycocalyx degradation. From analyzing clinical samples, we correlated glycocalyx degradation to MIF secretion. By applying the results from other studies, we hypothesized that HPA-1 or MMP-9 was involved in MIF-mediated glycocalyx degradation in dengue. This hypothesis was examined via *in vitro* experiments, which were carried out by recombinant NS1 stimulation, as it was indicated as an important effector in severe dengue. Since the interaction between different cell types is critical under physiological conditions, we assessed the DENV NS1-induced effects on both endothelial cells and leukocytes. To further elucidate the involvement of MMPs in this mechanism, MMP antibody array and gelatin zymography assays were performed. Subsequently, recombinant NS1 was injected into mice systemically or locally to confirm the involvement of MIF, HPA-1 and MMP-9 in NS1-induced endothelial glycocalyx degradation and hyperpermeability *in vivo*.

### Ethics statement

All research involving adult participants has been approved by the Institutional Review Board of NCKUH (IRB #B-ER-104-228). Informed written consent was not obtained from patients because the demographic and clinical information for the patients were delinked prior to analysis.

All animal studies were performed in accordance with the Guide for the Care and Use of Laboratory Animals (The Chinese-Taipei Society of Laboratory Animal Sciences, 2010) and were approved by the Institutional Animal Care and Use Committee (IACUC) of NCKU under the number IACUC 105018.

### Patient samples

In this study, serum samples were collected at Clinical Virology Laboratory of NCKUH from dengue patients in the acute stage (days 0–7 after illness onset) of the disease during a DENV outbreak in Tainan, Taiwan, in 2015 [54]. All dengue patient samples were screened via a rapid combo test for NS1 antigen and antibody detection and were assessed by qRT-PCR to quantify the DENV viral load. Patients were categorized as having dengue with warning signs or severe dengue according to the 2009 WHO criteria for dengue severity. The characteristics of these clinical samples are shown in S1 Table. In addition, 26 serum samples from healthy donors were included as the negative control.

### Recombinant proteins

Two different commercialized recombinant DENV serotype 2 NS1 proteins were used: one was produced from mammalian HEK 293T cells (The Native Antigen Company, Oxfordshire, UK), and the second was produced from drosophila S2 cells (CTK biotech, San Diego, CA, USA). These proteins were tested for endotoxin concentration by the Limulus amebocyte lysate (LAL) assay using the LAL Chromogenic Endotoxin Kit (Thermo Fisher Scientific, Waltham, MA, USA) and were shown to be endotoxin-free (<0.1 EU/ml). NS1 (20 μg/ml) from HEK 293T cells was used for *in vitro* experiments, and NS1 (50 μg/mouse) from S2 cells was used for the *in vivo* mice model.

DENV E domain III and prM proteins were cloned from DENV serotype 2 (strain PL046) using specific primers (for E domain III, forward: 5’-CATATGCGTTGCATAGGAATATCAAA-3’, reverse: 5’-CTCGAGTCCTCTGTCTACCATGGAGT-3’; and for prM, forward: 5’-CATATGTTCCATTTAACCACACGTAACG-3’, reverse: 5’-CTCGAGTCTTTTCTCTCTTCTGTGTTCT-3’). These proteins were cloned, expressed and purified from *E*. *coli* using Sepharose (GE Healthcare, Chicago, IL, USA), chelated with 500 mM cobalt chloride, and then slowly dialyzed against PBS.

Human MIF recombinant proteins were produced as previously described [26]. Briefly, human MIF proteins were cloned, expressed in *E*. *coli*, and purified by Sepharose (GE Healthcare). Heparan sulfate and thrombin with protease activity were purchased from Sigma-Aldrich (St. Louis, MO, USA) and were used in several studies [55, 56].

#### Inhibitors

To inhibit MIF activity, HUVECs were cotreated for 24 h with NS1 and either 100 μM p425 (6,6ʹ-[(3,3-dimethoxy[1,1ʹ-biphenyl]-4,4ʹ-diyl)bis(azo)]bis[4-amino-5-hydroxy-1,3-napthalenedisulphonic acid] tetrasodium salt; Calbiochem, La Jolla, CA, USA) or 50 μM ISO-1 ((S,R)-3-(4-hydroxyphenyl)-4,5-dihydro-5-isoxazole acetic acid; Calbiochem). In addition, a rabbit anti-MIF polyclonal antibody (10 μg/ml) was used in this study and was purified from recombinant MIF-immunized rabbit serum using a protein G affinity column (GE Healthcare), as previously described [26]. To inhibit HPA-1, OGT 2115 (Tocris Bioscience, Bristol, UK) was used at the indicated concentration. To inhibit MMP-9, SB-3CT (Abcam, Cambridge, UK) and MMP-9 inhibitor I (Santa Cruz, Dallas, TX, USA) were used at the indicated concentrations. Control mouse and rabbit IgGs were purchased from LeadGene Biomedical (Taiwan).

### Cells

HUVECs (Bioresource Collection and Research Center, Taiwan) were cultured in EGM-2 (Lonza, Basel, Switzerland), and THP-1 human monocytes (Bioresource Collection and Research Center, Taiwan) were cultured in Roswell Park Memorial Institute 1640 Medium (RPMI 1640; Thermo Fisher Scientific). Medium used to grow both cell types was supplemented with 10% fetal bovine serum (FBS; HyClone Laboratory, Logan, UT, USA), and cells were cultured at 37°C in a 5% CO_2_ atmosphere.

Human leukocytes (WBCs) were isolated from the whole blood of healthy donors. After collecting the blood into EDTA-containing plasma tubes, the whole blood was centrifuged at 1000 g for 5 min. The buffy coat was then collected and treated with red blood cell lysis buffer (Sigma-Aldrich, St. Louis, MO, USA). After one wash with PBS, the cells were cultured in serum-free RPMI 1640 at 37°C in a 5% CO_2_ atmosphere.

Human PBMCs were isolated from the whole blood of healthy donors using Ficoll-Paque (Sigma-Aldrich) according to the manufacturer’s instructions. Briefly, blood was collected into EDTA-containing vacutainers (BD, Franklin Lakes, NJ) and transferred to the top layer of Ficoll-Paque. After centrifugation at 2500 g for 30 min, the PBMCs were collected and washed with RPMI 1640 twice, and then cultured in RPMI 1640 containing 10% FBS at 37°C in a 5% CO_2_ atmosphere.

Stable MIF or Luc THP-1 knockdown THP-1 cells were generated as described in a previous study [57]. In brief, lentiviruses were generated from shRNA plasmids (MIF: TRCN0000056818; Luc: TRCN0000072243; National RNAi Core Facility, Academia Sinica, Taipei, Taiwan), and pMD.G and pCMVDR8.91 were cotransfected into HEK 293T cells (American Type Culture Collection, Manassas, VA, USA). THP-1 cells were infected with lentivirus and underwent selection in culture medium containing puromycin (1 μg/ml, Sigma-Aldrich).

### NS1 stimulation of THP-1 cells, human leukocytes and PBMCs

THP-1 cells were suspended in medium containing 5 ng/ml PMA (Sigma-Aldrich). After 16 h, THP-1 cells were resuspended in fresh medium without PMA and incubated for another 8 h. NS1 (20 μg/ml) was used to stimulate THP-1 cells, human leukocytes and PBMCs, and the resultant culture supernatants were collected at the indicated time points.

### Transwell permeability assay

A Transwell permeability assay was performed as described in a previous study [58]. HUVECs (2 x 10^5^) were grown on a Transwell insert (0.4 μm; Corning Life Sciences, Corning, NY, USA) until a monolayer formed. The upper chambers were reconstituted with 20 μg/ml NS1, culture supernatant from NS1-activated THP-1 cells, or the inhibitor-containing medium. After 24 h, the upper chambers were reconstituted with 300 μl of serum-free media containing 4.5 μl of streptavidin-horseradish peroxidase (HRP; R&D Systems, Inc., Minneapolis, MN, USA). Next, 20 μl of medium in the lower chamber was collected 5 min after the addition of streptavidin-HRP and was assayed for HRP activity by the addition of 100 μl of 3,3',5,5'-tetramethylbenzidine (TMB) substrate (R&D Systems). The color development at 450 nm was measured with a VersaMax microplate reader (Molecular Devices, Sunnyvale, CA, USA).

### Immunofluorescence staining

HUVECs were seeded as a monolayer onto a microscope cover glass slide and cultured under different conditions. After treatment for indicated time, the cells were fixed in 2% paraformaldehyde and then blocked with Superblock T20 (PBS) blocking buffer (Thermo Fisher Scientific).To measure the integrity of the endothelial glycocalyx and the deposition of CD138, the expression of sialic acid was stained with wheat germ agglutinin (WGA) lectin conjugated to FITC (WGA-FITC, Genetex) and the distribution of HPA-1 and CD138 was detected by anti-mouse-CD138 mAb (BD, Franklin Lakes, NJ, USA) or rabbit anti-HPA-1 polyclonal antibody (GeneTex). Primary antibodies were incubated with the fixed monolayer overnight at 4°C, followed by incubation with Alexa 488-conjugated goat anti-mouse IgG secondary antibody, Alexa 594-conjugated goat anti-rabbit IgG secondary antibody (Invitrogen, Carlsbad, CA, USA) (1:500 diluted) and Hoechst 33342 (Invitrogen, Carlsbad, CA, USA) (1:3,000 diluted) for 1 h. Images were captured using a confocal microscope (Olympus FluoView FV1000, Melville, NY, USA).

### Human MMP antibody array

The human MMP antibody array (Abcam) was used according to the manufacturer’s instructions. Briefly, array membranes were incubated in equal quantities of the culture supernatant from PBS- or NS1-treated THP-1 cells or NS1-treated leukocytes for 24 h overnight at 4°C. After washing with commercial wash buffer, the membranes were incubated with biotin-conjugated anti-MMP antibodies, followed by HRP-conjugated streptavidin. Bound HRP-conjugated antibodies were detected using the Luminata Crescendo Western HRP substrate (Merck Millipore, Darmstadt, Germany).

### Gelatin zymography assay

MMP activity in the culture supernatant was assayed by gelatin zymography using 7.5% acrylamide gel containing gelatin [59]. Briefly, the culture supernatant of NS1-treated THP-1 cells or leukocytes was concentrated. Non-heat-concentrated culture medium samples were mixed with nonreducing sample dye and electrophoresed at 120 V for 90 min. The gels were subsequently renatured and developed before being stained with Coomassie blue to reveal the positions of active gelatinases (clear bands) against the undigested gelatin substrate in the gel.

### DENV NS1-induced MIF, HPA-1, MMP-9 and CD138 secretion in mice

Mice were obtained from the animal center of NCKU. Before the injection of PBS or recombinant NS1, blood from 8- to 12-week-old BALB/c mice was collected by orbital sinus sampling with 10% citrate. Next, the mice were intravenously injected with 50 μg of NS1 or 100 μl of PBS. After the intravenous injection, blood from the mice was immediately collected by orbital sinus sampling and every 24 h thereafter until 120 h after the injection. The plasma concentrations of NS1, MIF, HPA-1, and MMP were analyzed by ELISA. For the peritoneal challenge, 500 μl of PBS, 50 μg of NS1, 50 μg of E or 50 μg of prM was injected intraperitoneally. After 24 h, the mice were sacrificed, and the abdominal cavities were washed with 5 ml of PBS. The resultant peritoneal lavage was collected, and the concentrations of MIF, HPA-1 and CD138 were quantified by ELISA.

### IHC staining of CD138 in mice

To further confirm that NS1 induced CD138 shedding in endothelial cells in mice, 50 μg of recombinant NS1, E or prM protein or 50 μl of PBS was subcutaneously injected into 8- to 12-week-old BALB/c mice, followed by a second injection of an equal amount of recombinant proteins or PBS 24 h after the first injection at the same site. The mice were sacrificed 24 h after the second injection. The separated skin tissues were fixed in formalin overnight and embedded in paraffin for the preparation of a series of sections. After paraffin removal and antigen retrieval by citrate buffer, the tissue sections were blocked, and immunohistochemistry was performed using the Mouse/Rabbit HRP Detection System with DAB (brown) (BioTnA Biotech, Kaohsiung, Taiwan). Hematoxylin was used as a counterstain. Anti-α-SMA antibody (Arigo, Hsinchu City, Taiwan) was used at 1:200, and anti-CD138 antibody (BD, Franklin Lakes, NJ) was used at 1:100. The resultant images were acquired using phase-contrast microscopy (Olympus, Tokyo, Japan).

### ELISA

The concentrations of MIF, HPA-1, CD138, MMP-9, IL-6 and IL-8 in the serum or cell culture medium were measured using human MIF, HPA-1, CD138, MMP-9, IL-6 and IL-8 ELISA kits (R&D Systems) following the manufacturer’s instructions. The concentrations of MIF, HPA-1, MMP-9 and CD138 in the serum or peritoneal lavage fluid of mice were measured using mouse MIF, HPA-1, MMP-9 and CD138 ELISA kits (BlueGene Biotech, Shanghai, China). NS1 ELISA was carried out using paired anti-NS1 antibodies prepared in our laboratory and was quantified by the addition of 100 μl of 3,3',5,5'-tetramethylbenzidine (TMB) substrate (R&D Systems).

### Statistical analysis

The patients’ sera data were expressed as the median ± interquartile range and tested if the values come from a Gaussian distribution by using D’Agostino and Pearson omnibus normality test. If the data meet Gaussian distribution, the significance of differences between each groups was analyzied using One-way ANOVA with Tukey’s method. If the data do not meet the assumptions of normality, they were analyzed with a non-parametric test by Kruskal-Wallis test. The *in vitro* and *in vivo* data are expressed as the mean ± standard deviation (SD) from more than three independent experiments. Student’s t-test was used to analyze the significance of differences between the test and control groups. One-way ANOVA with Kruskal-Wallis comparison test was used to analyze the significance of differences between multiple groups. All data were analyzed by GraphPad Prism 5 software. P values <0.05 were considered statistically significant.

## Supporting information

S1 Table Characteristics of dengue patients. (DOCX)

S1 Fig The correlations of serum NS1, HPA-1, MMP-9, CD138, MIF levels and viral load in severe dengue patients. The correlations of the concentrations of **(A)** NS1, **(B)** HPA-1, **(C)** MMP-9, **(D)** CD138 and **(E)** MIF and viral load in the same group of severe dengue patients were plotted. Linear regressions were analyzed using nonparametric correlation test (panel A, B, C, D and E).
(DOCX)

S2 Fig DENV NS1 induces HPA-1 activation and vascular leakage. **(A)** HUVECs were treated with PBS, NS1 or NS1 mixed with anti-NS1 antibodies (2E8) or control mouse IgG (cmIgG) for 24 h, and the HPA-1 level was determined by western blot. The relative HPA-1 protein level (including the proform and active form) was normalized to β-actin, and the fold change is noted under each band. **(B)** BALB/c mice were intravenously injected with Evans Blue dye, followed by subcutaneous injections of PBS or different doses of HPA-1, heat-denatured HPA-1 or thrombin. After the mice were sacrificed, skin samples were collected and processed 3 h postinjection.
(DOCX)

S3 Fig DENV NS1-induced MIF secretion causes glycocalyx degradation and hyperpermeability in HUVECs. **(A)** HUVECs were treated with different concentrations of NS1 for the indicated times, followed supernatant collection for the detection of MIF by ELISA. **(B)** HUVEC monolayers were incubated with the supernatant from control or NS1-treated HUVEC cultures for the indicated times, and the endothelial permeability was then determined by Transwell permeability assay. **(C)** HUVEC monolayers were incubated with control or NS1-treated HUVEC-conditioned medium for 24 h. The concentration of CD138 in the supernatant after the incubation was determined by ELISA. **(D) (E)** Control or NS1-treated HUVEC culture supernatant with or without anti-MIF polyclonal antibodies or HPA-1 inhibitor (OGT 2115) or anti-NS1 antibodies (2E8) and incubated with HUVECs for 24 h. **(D)** The endothelial permeability was determined by Transwell permeability assay, and **(E)** the concentration of CD138 in the supernatant was determined by ELISA. **(F)** HUVEC monolayers were treated with PBS, NS1 or NS1 mixed with anti-NS1 antibodies (2E8) or HPA-1 inhibitor (OGT 2115) for the indicated times, and the concentration of MIF in the supernatant was determined by ELISA; S/N, supernatant; *P<0.05, **P<0.005, ***P<0.001; unpaired t-test (panel B and C), Kruskal-Wallis ANOVA (panel D and E).
(DOCX)

S4 Fig MIF induces HPA-1 activation and glycocalyx shedding in HUVECs. **(A)** HUVECs were treated with or without MIF recombinant protein (1 μg/ml) for the indicated times, and the concentration of CD138 in the supernatant was determined by ELISA. **(B)** HUVECs were treated with or without MIF recombinant protein (1 μg/ml) for 18 h, and the HPA-1 level was determined by western blot. The relative HPA-1 protein level (including the proform and active form) was normalized to β-actin, and the fold change is noted under each band. **(C)** HUVECs were treated as indicated for 18 h and then stained for HPA-1 (red), CD138 (green), and nuclei (blue). *P<0.05, **P<0.005; unpaired t-test (panel A).
(DOCX)

S5 Fig DENV NS1 does not induce MIF and MMP-9 secretion in PBMCs. **(A) (B)** Isolated human PBMCs were treated with or without NS1 (10 μg/ml) for the indicated times, and the concentration of **(A)** MIF and **(B)** MMP-9 in the supernatant was determined by ELISA.
(DOCX)

S6 Fig DENV NS1-induced MMP-9 secretion from WBCs causes endothelial hyperpermeability. Isolated human WBCs were treated with or without NS1 for 24 h, and the supernatants were collected. HUVEC monolayers were incubated with the supernatant from control or NS1-treated WBCs for 6 h; then, endothelial permeability was determined by Transwell permeability assay. S/N, supernatant; *P<0.05; Kruskal-Wallis ANOVA.
(DOCX)

S7 Fig Cytokine secretion profile of WBCs and THP-1 cells after DENV NS1 stimulation. **(A) (B) (C)** Isolated human WBCs and **(D) (E) (F)** THP-1 cells were treated with or without NS1 for the indicated times, and the concentration of MIF, IL-6 and IL-8 in the supernatant was determined by ELISA; *P<0.05, **P<0.005, ***P<0.001; unpaired t-test (panel A, B, C and D).
(DOCX)

S8 Fig DENV NS1-induced endothelial hyperpermeability is mediated by MIF. **(A)** HUVECs were transfected with MIF shRNA (shMIF) or scrambled shRNA (shLuc). The cell lysates were collected, and the relative protein level of MIF was measured by western blot. **(B)** The permeability of shMIF HUVECs and shLuc HUVECs after 24 h of NS1 treatment was detected by Transwell permeability assay. The results are presented as the mean ± SD of triplicate measurements.
(DOCX)

S9 Fig Inhibition of MIF and MMP-9 attenuate NS1-induced vascular leakage in mice. BALB/c mice were intravenously injected with Evans Blue dye, followed by the subcutaneous injection of PBS or different doses of NS1, NS1 with MMP-9 inhibitor I or NS1 with ISO-1 for 6 h. After 5 h, the mice were subcutaneously injected with thrombin as a positive control. After another hour, the mice were sacrificed, and skin samples were collected and processed.
(DOCX)

S10 Fig The correlations of serum levels of HPA-1 with NS1 and MIF in severe dengue patients. **(A)** The correlations of the concentrations of **(A)** NS1, **(B)** MIF, and HPA-1 in the severe dengue patients were plotted. Linear regressions were analyzed using nonparametric correlation test (panel A and B).
(DOCX)
