# Supplementary figures and images for: Macrophage migration inhibitory factor is critical for dengue NS1-induced endothelial glycocalyx degradation and hyperpermeability
Source: PLoS Pathog. 2018 Apr 27;14(4):e1007033. doi: 10.1371/journal.ppat.1007033 (PMC6044858; doi:10.1371/journal.ppat.1007033)

**
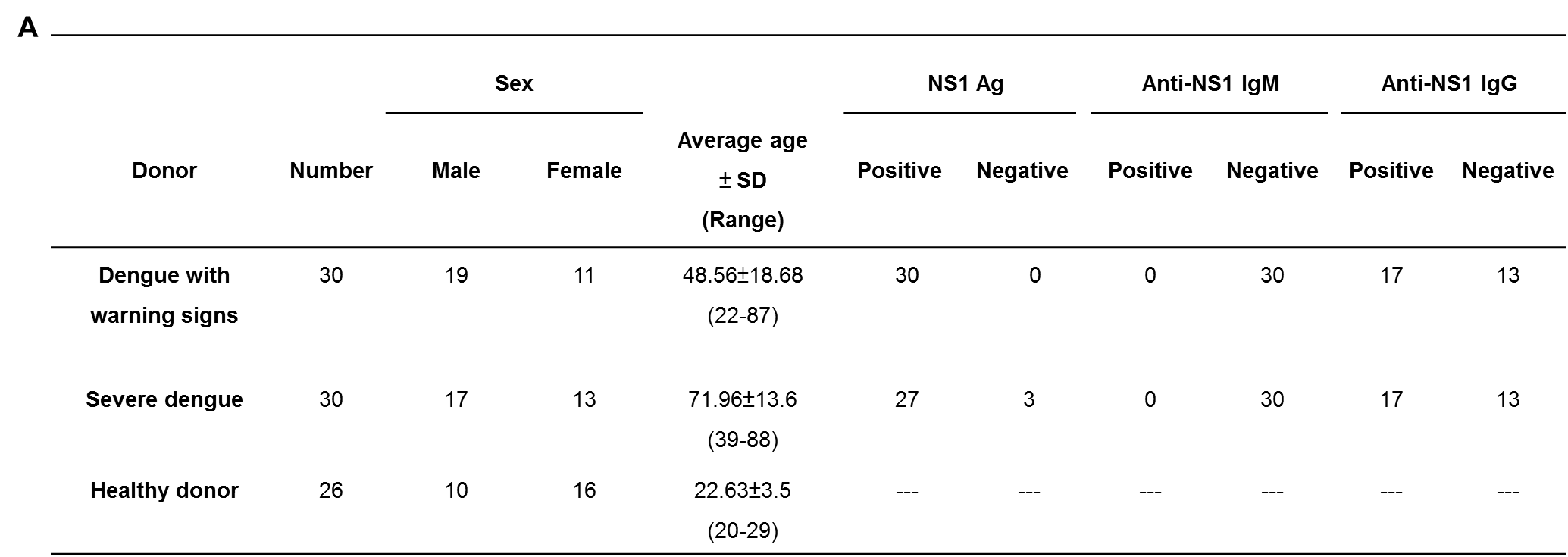
S1 Table. Characteristics of dengue patients**

Supplement: S1 Table — (DOCX) [file ppat.1007033.s001.docx]
