# Supplementary material for: Macrophage migration inhibitory factor is critical for dengue NS1-induced endothelial glycocalyx degradation and hyperpermeability
Source: PLoS Pathog. 2018 Apr 27;14(4):e1007033. doi: 10.1371/journal.ppat.1007033 (PMC6044858; doi:10.1371/journal.ppat.1007033)
Supplement: S1 Fig — The correlations of the concentrations of (A) NS1, (B) HPA-1, (C) MMP-9, (D) CD138 and (E) MIF and viral load in the same group of severe dengue patients were plotted. Linear regressions were analyzed using nonparametric correlation test (panel A, B, C, D and E). (DOCX) [file ppat.1007033.s002.docx]

**
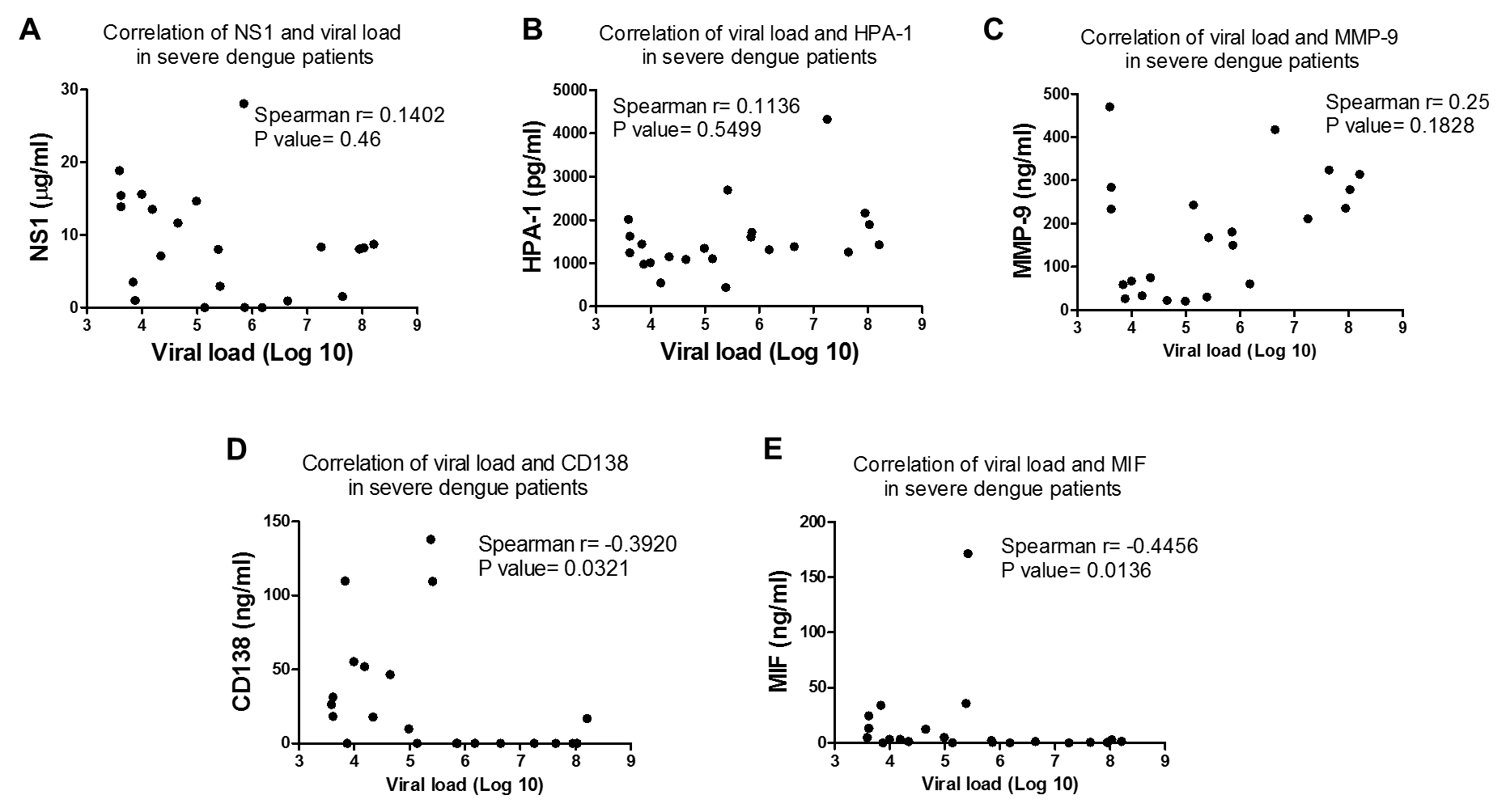
S1 Fig. The correlations of serum NS1, HPA-1, MMP-9, CD138, MIF levels and viral load in severe dengue patients**. The correlations of the concentrations of **(A)** NS1, **(B)** HPA-1, **(C)** MMP-9, **(D)** CD138 and **(E)** MIF and viral load in the same group of severe dengue patients were plotted. Linear regressions were analyzed using nonparametric correlation test (panel A, B, C, D and E).
