# Supplementary material for: Macrophage migration inhibitory factor is critical for dengue NS1-induced endothelial glycocalyx degradation and hyperpermeability
Source: PLoS Pathog. 2018 Apr 27;14(4):e1007033. doi: 10.1371/journal.ppat.1007033 (PMC6044858; doi:10.1371/journal.ppat.1007033)
Supplement: S2 Fig — (A) HUVECs were treated with PBS, NS1 or NS1 mixed with anti-NS1 antibodies (2E8) or control mouse IgG (cmIgG) for 24 h, and the HPA-1 level was determined by western blot. The relative HPA-1 protein level (including the proform and active form) was normalized to β-actin, and the fold change is noted under each band. (B) BALB/c mice were intravenously injected with Evans Blue dye, followed by subcutaneous injections of PBS or different doses of HPA-1, heat-denatured HPA-1 or thrombin. After the mice were sacrificed, skin samples were collected and processed 3 h postinjection. (DOCX) [file ppat.1007033.s003.docx]

**
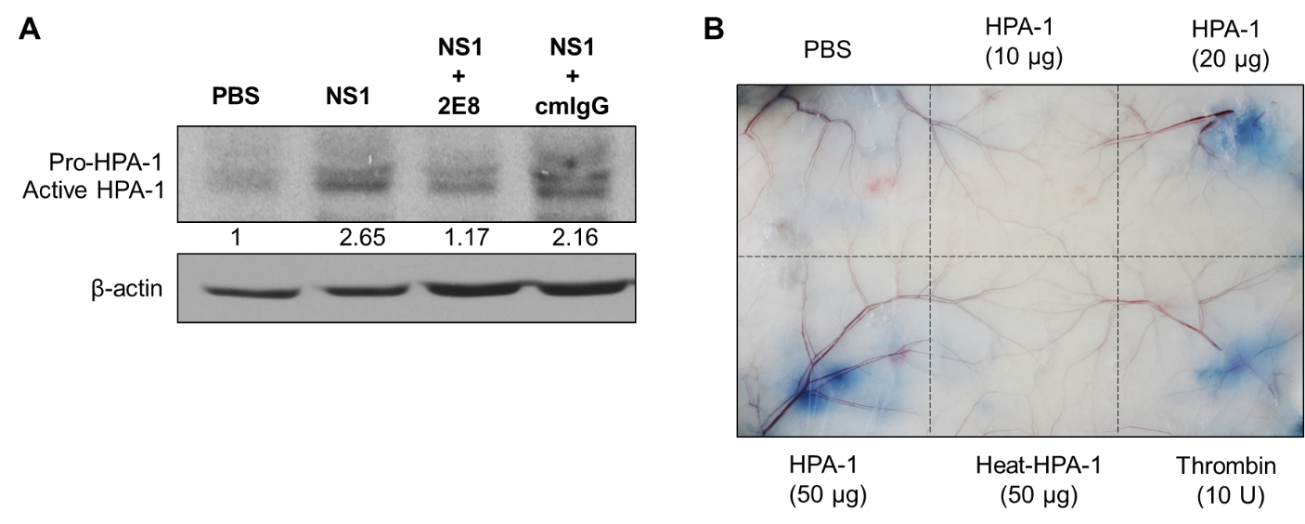
**

**S2 Fig. DENV NS1 induces HPA-1 activation and vascular leakage.** **(A)** HUVECs were treated with PBS, NS1 or NS1 mixed with anti-NS1 antibodies (2E8) or control mouse IgG (cmIgG) for 24 h, and the HPA-1 level was determined by western blot. The relative HPA-1 protein level (including the proform and active form) was normalized to β-actin, and the fold change is noted under each band. **(B)** BALB/c mice were intravenously injected with Evans Blue dye, followed by subcutaneous injections of PBS or different doses of HPA-1, heat-denatured HPA-1 or thrombin. After the mice were sacrificed, skin samples were collected and processed 3 h postinjection.
