# Supplementary material for: Macrophage migration inhibitory factor is critical for dengue NS1-induced endothelial glycocalyx degradation and hyperpermeability
Source: PLoS Pathog. 2018 Apr 27;14(4):e1007033. doi: 10.1371/journal.ppat.1007033 (PMC6044858; doi:10.1371/journal.ppat.1007033)
Supplement: S3 Fig — (A) HUVECs were treated with different concentrations of NS1 for the indicated times, followed supernatant collection for the detection of MIF by ELISA. (B) HUVEC monolayers were incubated with the supernatant from control or NS1-treated HUVEC cultures for the indicated times, and the endothelial permeability was then determined by Transwell permeability assay. (C) HUVEC monolayers were incubated with control or NS1-treated HUVEC-conditioned medium for 24 h. The concentration of CD138 in the supernatant after the incubation was determined by ELISA. (D) (E) Control or NS1-treated HUVEC culture supernatant with or without anti-MIF polyclonal antibodies or HPA-1 inhibitor (OGT 2115) or anti-NS1 antibodies (2E8) and incubated with HUVECs for 24 h. (D) The endothelial permeability was determined by Transwell permeability assay, and (E) the concentration of CD138 in the supernatant was determined by ELISA. (F) HUVEC monolayers were treated with PBS, NS1 or NS1 mixed with anti-NS1 antibodies (2E8) or HPA-1 inhibitor (OGT 2115) for the indicated times, and the concentration of MIF in the supernatant was determined by ELISA; S/N, supernatant; *P<0.05, **P<0.005, ***P<0.001; unpaired t-test (panel B and C), Kruskal-Wallis ANOVA (panel D and E). (DOCX) [file ppat.1007033.s004.docx]

**
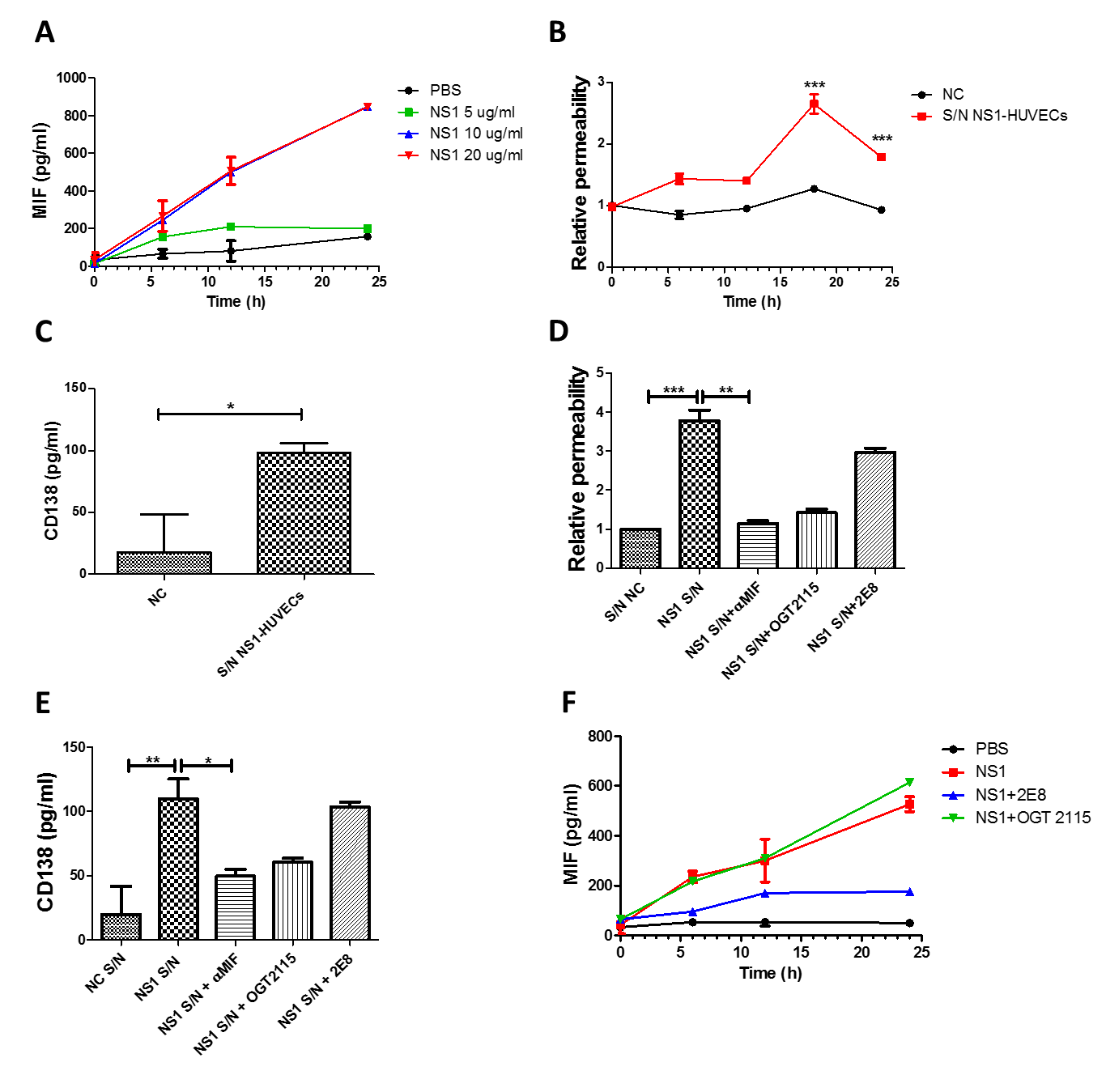
**

**S3 Fig. DENV NS1-induced MIF secretion causes glycocalyx degradation and hyperpermeability in HUVECs. (A)** HUVECs were treated with different concentrations of NS1 for the indicated times, followed supernatant collection for the detection of MIF by ELISA. **(B)** HUVEC monolayers were incubated with the supernatant from control or NS1-treated HUVEC cultures for the indicated times, and the endothelial permeability was then determined by Transwell permeability assay. **(C)** HUVEC monolayers were incubated with control or NS1-treated HUVEC-conditioned medium for 24 h. The concentration of CD138 in the supernatant after the incubation was determined by ELISA. **(D) (E)** Control or NS1-treated HUVEC culture supernatant with or without anti-MIF polyclonal antibodies or HPA-1 inhibitor (OGT 2115) or anti-NS1 antibodies (2E8) and incubated with HUVECs for 24 h. **(D)** The endothelial permeability was determined by Transwell permeability assay, and **(E)** the concentration of CD138 in the supernatant was determined by ELISA. **(F)** HUVEC monolayers were treated with PBS, NS1 or NS1 mixed with anti-NS1 antibodies (2E8) or HPA-1 inhibitor (OGT 2115) for the indicated times, and the concentration of MIF in the supernatant was determined by ELISA; S/N, supernatant; *P<0.05, **P<0.005, ***P<0.001; unpaired t-test (panel B and C), Kruskal-Wallis ANOVA (panel D and E).
