# Supplementary material for: Macrophage migration inhibitory factor is critical for dengue NS1-induced endothelial glycocalyx degradation and hyperpermeability
Source: PLoS Pathog. 2018 Apr 27;14(4):e1007033. doi: 10.1371/journal.ppat.1007033 (PMC6044858; doi:10.1371/journal.ppat.1007033)
Supplement: S4 Fig — (A) HUVECs were treated with or without MIF recombinant protein (1 μg/ml) for the indicated times, and the concentration of CD138 in the supernatant was determined by ELISA. (B) HUVECs were treated with or without MIF recombinant protein (1 μg/ml) for 18 h, and the HPA-1 level was determined by western blot. The relative HPA-1 protein level (including the proform and active form) was normalized to β-actin, and the fold change is noted under each band. (C) HUVECs were treated as indicated for 18 h and then stained for HPA-1 (red), CD138 (green), and nuclei (blue). *P<0.05, **P<0.005; unpaired t-test (panel A). (DOCX) [file ppat.1007033.s005.docx]

**
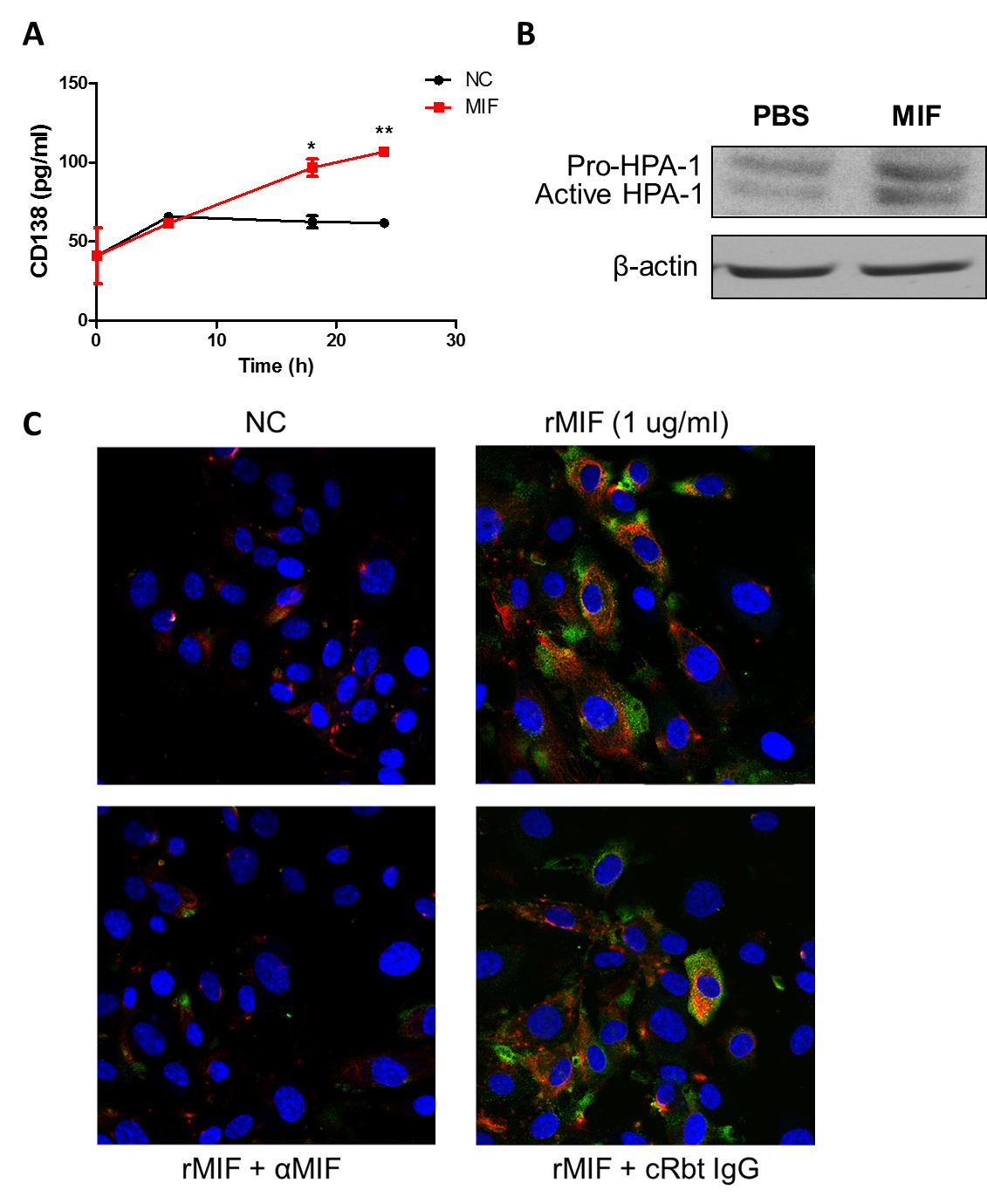
**

**S4 Fig. MIF induces HPA-1 activation and glycocalyx shedding in HUVECs.** **(A)** HUVECs were treated with or without MIF recombinant protein (1 μg/ml) for the indicated times, and the concentration of CD138 in the supernatant was determined by ELISA. **(B)** HUVECs were treated with or without MIF recombinant protein (1 μg/ml) for 18 h, and the HPA-1 level was determined by western blot. The relative HPA-1 protein level (including the proform and active form) was normalized to β-actin, and the fold change is noted under each band. **(C)** HUVECs were treated as indicated for 18 h and then stained for HPA-1 (red), CD138 (green), and nuclei (blue). *P<0.05, **P<0.005; unpaired t-test (panel A).
