# Supplementary material for: Macrophage migration inhibitory factor is critical for dengue NS1-induced endothelial glycocalyx degradation and hyperpermeability
Source: PLoS Pathog. 2018 Apr 27;14(4):e1007033. doi: 10.1371/journal.ppat.1007033 (PMC6044858; doi:10.1371/journal.ppat.1007033)
Supplement: S5 Fig — (A) (B) Isolated human PBMCs were treated with or without NS1 (10 μg/ml) for the indicated times, and the concentration of (A) MIF and (B) MMP-9 in the supernatant was determined by ELISA. (DOCX) [file ppat.1007033.s006.docx]

**
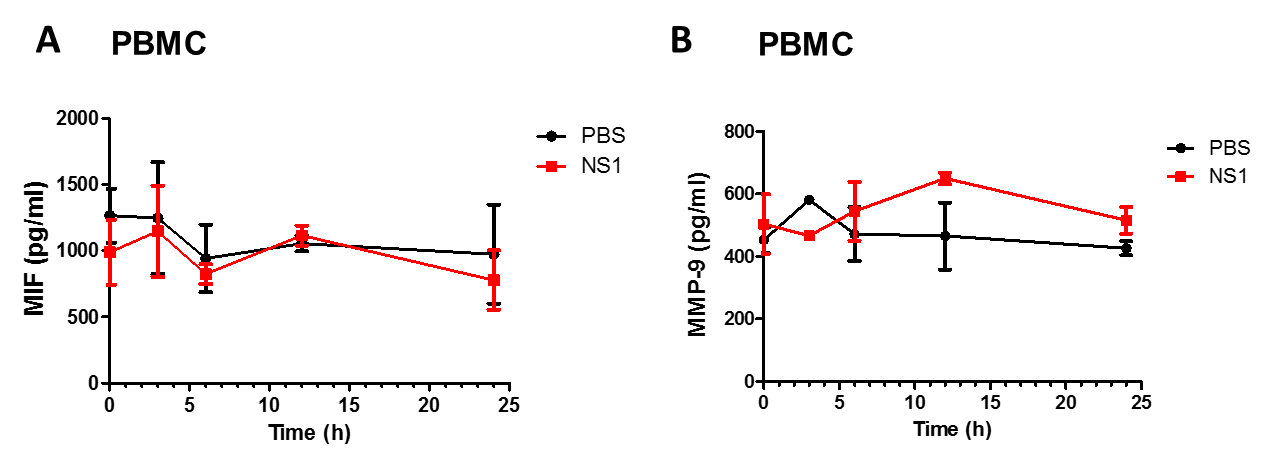
**

**S5 Fig. DENV NS1 does not induce MIF and MMP-9 secretion in PBMCs. (A) (B)** Isolated human PBMCs were treated with or without NS1 (10 μg/ml) for the indicated times, and the concentration of **(A)** MIF and **(B)** MMP-9 in the supernatant was determined by ELISA.
