# Supplementary material for: Macrophage migration inhibitory factor is critical for dengue NS1-induced endothelial glycocalyx degradation and hyperpermeability
Source: PLoS Pathog. 2018 Apr 27;14(4):e1007033. doi: 10.1371/journal.ppat.1007033 (PMC6044858; doi:10.1371/journal.ppat.1007033)
Supplement: S6 Fig — Isolated human WBCs were treated with or without NS1 for 24 h, and the supernatants were collected. HUVEC monolayers were incubated with the supernatant from control or NS1-treated WBCs for 6 h; then, endothelial permeability was determined by Transwell permeability assay. S/N, supernatant; *P<0.05; Kruskal-Wallis ANOVA. (DOCX) [file ppat.1007033.s007.docx]

**
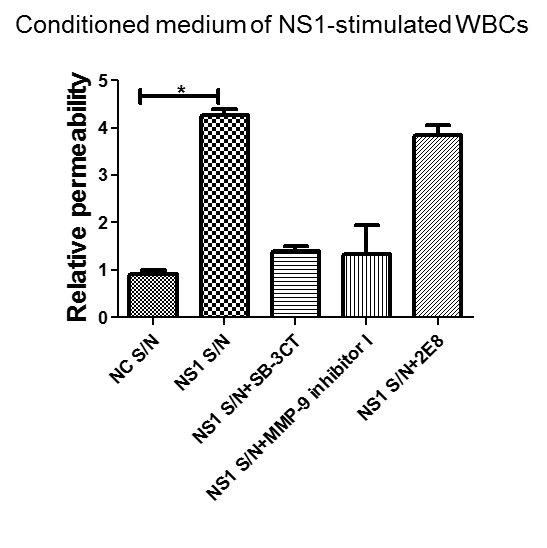
**

**S6 Fig. DENV NS1-induced MMP-9 secretion from WBCs causes endothelial hyperpermeability.** Isolated human WBCs were treated with or without NS1 for 24 h, and the supernatants were collected. HUVEC monolayers were incubated with the supernatant from control or NS1-treated WBCs for 6 h; then, endothelial permeability was determined by Transwell permeability assay. S/N, supernatant; *P<0.05; Kruskal-Wallis ANOVA.
