# Supplementary material for: Macrophage migration inhibitory factor is critical for dengue NS1-induced endothelial glycocalyx degradation and hyperpermeability
Source: PLoS Pathog. 2018 Apr 27;14(4):e1007033. doi: 10.1371/journal.ppat.1007033 (PMC6044858; doi:10.1371/journal.ppat.1007033)
Supplement: S7 Fig — (A) (B) (C) Isolated human WBCs and (D) (E) (F) THP-1 cells were treated with or without NS1 for the indicated times, and the concentration of MIF, IL-6 and IL-8 in the supernatant was determined by ELISA; *P<0.05, **P<0.005, ***P<0.001; unpaired t-test (panel A, B, C and D). (DOCX) [file ppat.1007033.s008.docx]

**
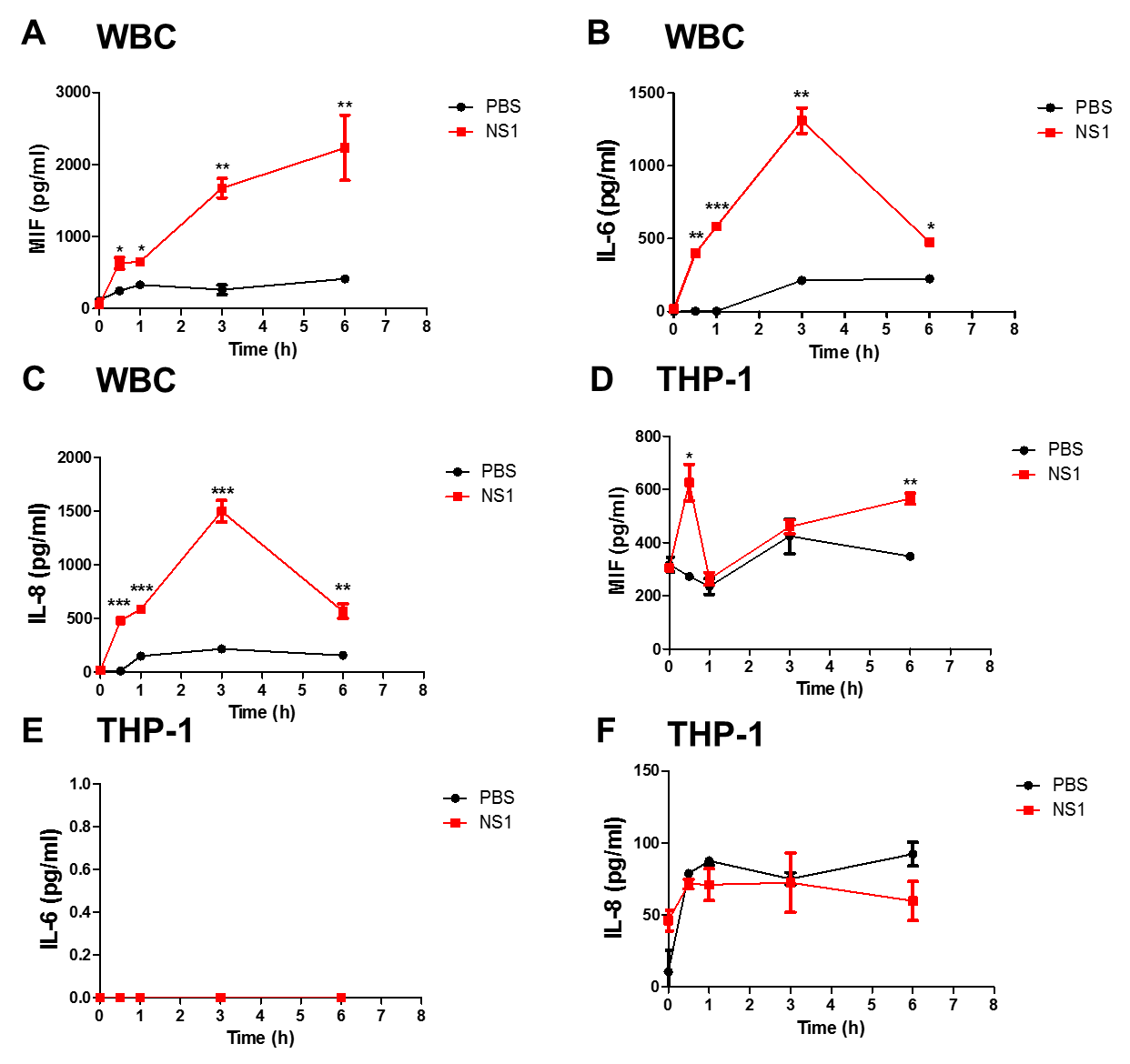
**

**S7 Fig. Cytokine secretion profile of WBCs and THP-1 cells after DENV NS1 stimulation. (A) (B) (C)** Isolated human WBCs and **(D) (E) (F)** THP-1 cells were treated with or without NS1 for the indicated times, and the concentration of MIF, IL-6 and IL-8 in the supernatant was determined by ELISA; *P<0.05, **P<0.005, ***P<0.001; unpaired t-test (panel A, B, C and D).
