# Supplementary material for: Macrophage migration inhibitory factor is critical for dengue NS1-induced endothelial glycocalyx degradation and hyperpermeability
Source: PLoS Pathog. 2018 Apr 27;14(4):e1007033. doi: 10.1371/journal.ppat.1007033 (PMC6044858; doi:10.1371/journal.ppat.1007033)
Supplement: S8 Fig — (A) HUVECs were transfected with MIF shRNA (shMIF) or scrambled shRNA (shLuc). The cell lysates were collected, and the relative protein level of MIF was measured by western blot. (B) The permeability of shMIF HUVECs and shLuc HUVECs after 24 h of NS1 treatment was detected by Transwell permeability assay. The results are presented as the mean ± SD of triplicate measurements. (DOCX) [file ppat.1007033.s009.docx]

**
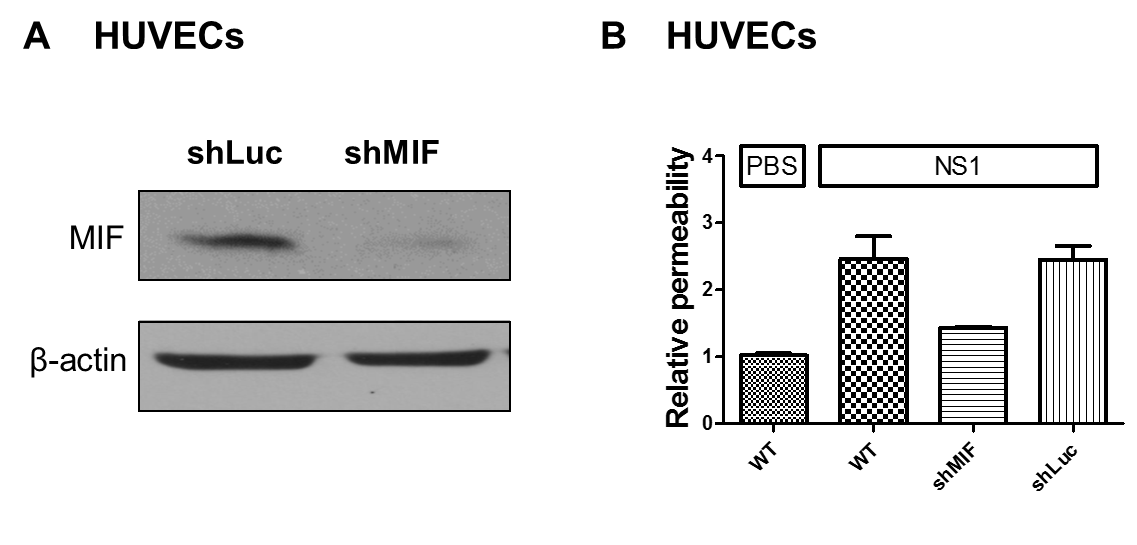
**

**S8 Fig. DENV NS1-induced endothelial hyperpermeability is mediated by MIF.
(A)** HUVECs were transfected with MIF shRNA (shMIF) or scrambled shRNA (shLuc). The cell lysates were collected, and the relative protein level of MIF was measured by western blot. **(B)** The permeability of shMIF HUVECs and shLuc HUVECs after 24 h of NS1 treatment was detected by Transwell permeability assay. The results are presented as the mean ± SD of triplicate measurements.
