# Supplementary material for: Macrophage migration inhibitory factor is critical for dengue NS1-induced endothelial glycocalyx degradation and hyperpermeability
Source: PLoS Pathog. 2018 Apr 27;14(4):e1007033. doi: 10.1371/journal.ppat.1007033 (PMC6044858; doi:10.1371/journal.ppat.1007033)
Supplement: S9 Fig — BALB/c mice were intravenously injected with Evans Blue dye, followed by the subcutaneous injection of PBS or different doses of NS1, NS1 with MMP-9 inhibitor I or NS1 with ISO-1 for 6 h. After 5 h, the mice were subcutaneously injected with thrombin as a positive control. After another hour, the mice were sacrificed, and skin samples were collected and processed. (DOCX) [file ppat.1007033.s010.docx]

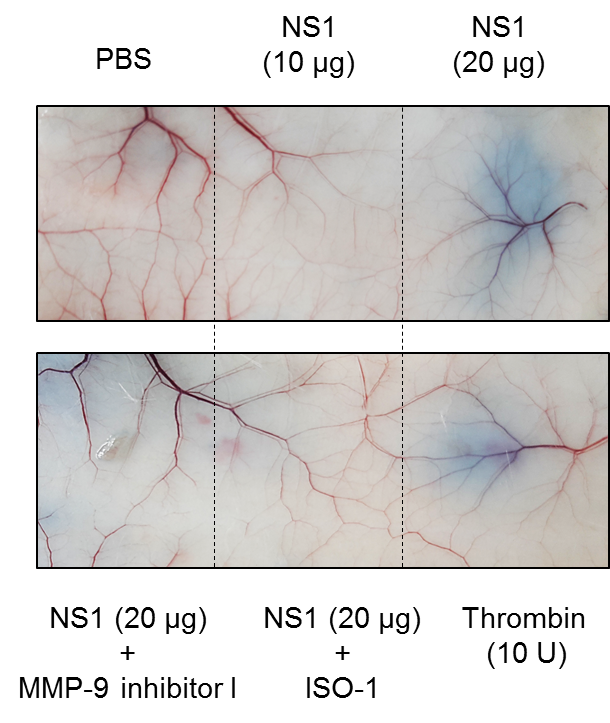


**S9 Fig. Inhibition of MIF and MMP-9 attenuated NS1-induced vascular leakage in mice.** BALB/c mice were intravenously injected with Evans Blue dye, followed by the subcutaneous injection of PBS or different doses of NS1, NS1 with MMP-9 inhibitor I or NS1 with ISO-1 for 6 h. After 5 h, the mice were subcutaneously injected with thrombin as a positive control. After another hour, the mice were sacrificed, and skin samples were collected and processed.
