# Supplementary material for: Macrophage migration inhibitory factor is critical for dengue NS1-induced endothelial glycocalyx degradation and hyperpermeability
Source: PLoS Pathog. 2018 Apr 27;14(4):e1007033. doi: 10.1371/journal.ppat.1007033 (PMC6044858; doi:10.1371/journal.ppat.1007033)
Supplement: S10 Fig — (A) The correlations of the concentrations of (A) NS1, (B) MIF, and HPA-1 in the severe dengue patients were plotted. Linear regressions were analyzed using nonparametric correlation test (panel A and B). (DOCX) [file ppat.1007033.s011.docx]

**
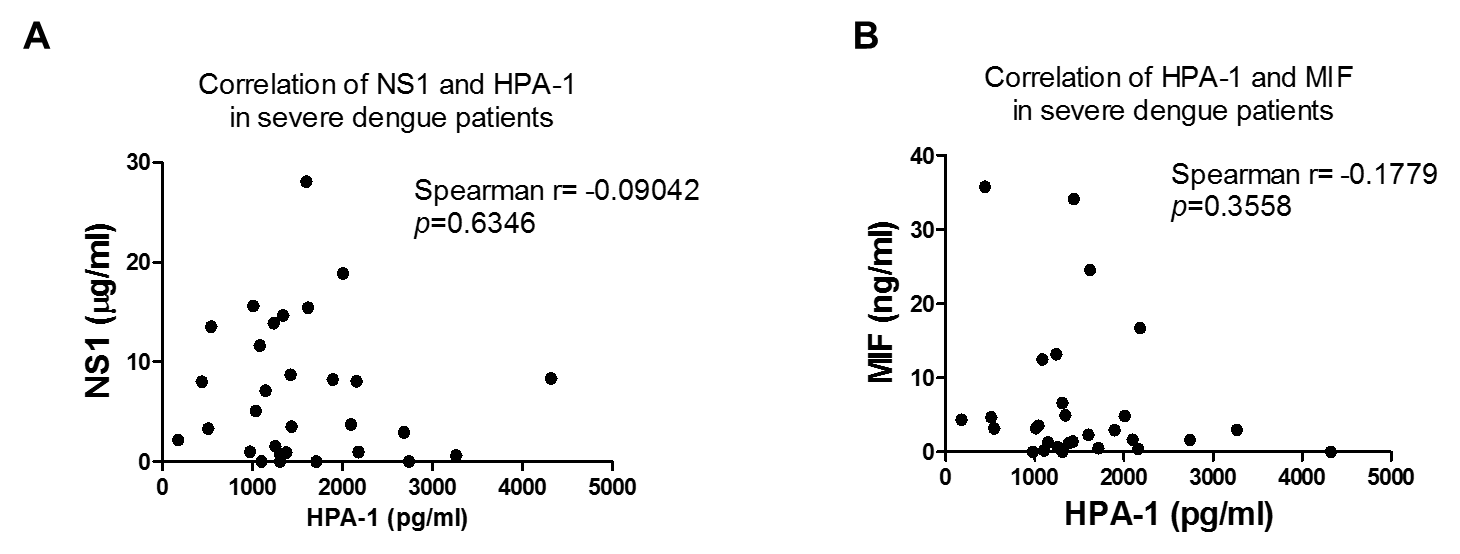
S10 Figure. The correlations of serum levels of HPA-1 with NS1 and MIF in severe dengue patients. (A)** The correlations of the concentrations of **(A)** NS1, **(B)** MIF, and HPA-1 in the severe dengue patients were plotted. Linear regressions were analyzed using nonparametric correlation test (panel A and B).
